# Stereoselective Total Synthesis of Natural Decanolides Bellidisin C and Pinolidoxin

**DOI:** 10.3390/molecules29235500

**Published:** 2024-11-21

**Authors:** Jingjing Bi, Minhao Chen, Pengpeng Nie, Yuanfang Liu, Jun Liu, Yuguo Du

**Affiliations:** 1 School of Pharmacy, Xinyang Agriculture and Forestry University, Xinyang 464000, China; 2 School of Chemistry and Chemical Engineering, Henan Normal University, Xinxiang 453007, China; 3 State Key Laboratory of Environmental Chemistry and Eco-Toxicology, Research Center for Eco-Environmental Sciences, Chinese Academy of Sciences, Beijing 100085, China; a17337704506@163.com (M.C.); niepengpeng20@mails.ucas.ac.cn (P.N.); yuanfangl@126.com (Y.L.); duyuguo@rcees.ac.cn (Y.D.); 4 School of Chemical Science, University of Chinese Academy of Sciences, Beijing 100049, China; 5 Binzhou Institute of Technology, Weiqiao-UCAS Science and Technology Park, Binzhou 256606, China

**Keywords:** decanolide, bellidisin C, pinolidoxin, total synthesis

## Abstract

A divergent total synthesis of bioactive, naturally occurring decanolides, pinolidoxin and bellidisin C, was accomplished by taking advantage of chiral templates *L*-ribose and *L*-malic acid. In particular, bellidisin C, which is the first total synthesis so far, was achieved through a cascade reaction of reductive elimination and nucleophilic addition in a one-pot process and a sodium–alkoxide-promoted intramolecular lactonization as the key steps.

## 1. Introduction

Naturally occurring decanolides (ten-membered lactones, TMLs) represent an interesting and structurally diverse group of secondary metabolites with potent and diverse biological activity [1,2,3]. Among these decanolides are important compounds such as herbarumins [4], microcarpalide [5], and stagonolide [6], which exhibited promising biological activities, including phytotoxic, antibacterial, antitumoral, and insecticidal properties. The broad spectrum of biological activities and diverse architectural features of these medium-ring lactones have attracted immense interest from the synthetic community [7,8,9,10].

**Figure 1:**
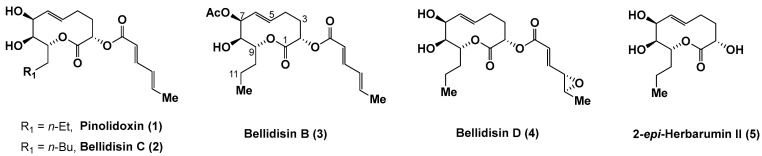
Structures of naturally occurring decanolides from *P. bellidi*.

Recently, three novel naturally occurring decanolides, named bellidisins B–D (Figure 1), were isolated from the ethyl acetate extraction of the rice fermentation of *Phoma bellidis* along with known phytotoxic metabolites pinolidoxin (**1**) [11,12,13,14,15,16] and 2-*epi*-herbarumin II (**5**) [17]. The bellidisins exhibited significant cytotoxicity against a panel of tumor cell lines, especially HL-60 and SW480 cell lines. The structures of the bellidisins B–D (**2–4**) were elucidated using extensive spectroscopic analysis and electronic circular dichroism (ECD) calculations [11]. The bellidisins B–D comprise similar decanolide skeletons, with an extra sorbate side chain connecting at C-2. Architecturally, the backbone and stereochemistry of the bellidisins are very close to the previously isolated pinolidoxin (**1**). Pinolidoxin was first isolated in 1993 from the fungus *Ascochyta pinodes* by Evidente et al. [12] and later, from an aggressive strain of *Didymella pinodes* isolated from infected pea in Spain by same group in 2012 [13]. A combination of extensive spectroscopic analysis and degradation experiments led Evidente [12,13] and Piccialli [14] to propose the structure of pinolidoxin (**1**), as shown in Figure 1, in which the absolute configuration at C-2 remained unclear due to limited supply. It is noteworthy that decanolide **1** was also isolated from a strain of the fungus *Mycosphaerella lethalis* by Arnone et al. in 1993 and named lethaloxin [15]. Preliminary biological studies indicated that pinolidoxin appeared to be potent inhibitors of phenylalanine ammonialyase (PAL) activity, a key regulatory enzyme in the phenylpropanoid metabolic pathway activated following the pathogen attack [12,13]. Very recently, Hitora et al. reported that pinolidoxin displayed an inhibitory effect on osteoclast differentiation and did not exhibit cytotoxicity on RAW264 cells [16].

To date, three syntheses of pinolidoxin have been disclosed, and no synthetic efforts have been reported toward the synthesis of bellidisins [18,19,20]. Pioneering synthetic studies of pinolidoxin/lethaloxin was conducted by Fürstner group [18]. In 2002, Fürstner and co-workers reported the first total synthesis and configuration assignment of (+)-pinolidoxin on the basis of dynamic calculations and single-crystal X-ray diffraction analysis. Soon after, Kozmin group reported the total synthesis of unnatural enantiomer (−)-pinolidoxin from meso silacyclic epoxides using their catalytic desymmetrization and a late-stage ring-closing metathesis (RCM) as the key steps [19]. In 2005, Marco and co-workers reported their synthesis of (+)-lethaloxin from (*R*)-glycidol and *D*-ribose in nine linear steps with 8.38% overall yield and thereby established that (+)-lethaloxin and (+)-pinolidoxin were identical [15,20]. In the context of our studies on the enantioselective synthesis of bioactive natural products based on a chiron approach [21,22,23,24,25], we herein report a concise and efficient synthetic approach to pinolidoxin and bellidisin C.

## 2. Results and Discussion

**Scheme 1:**
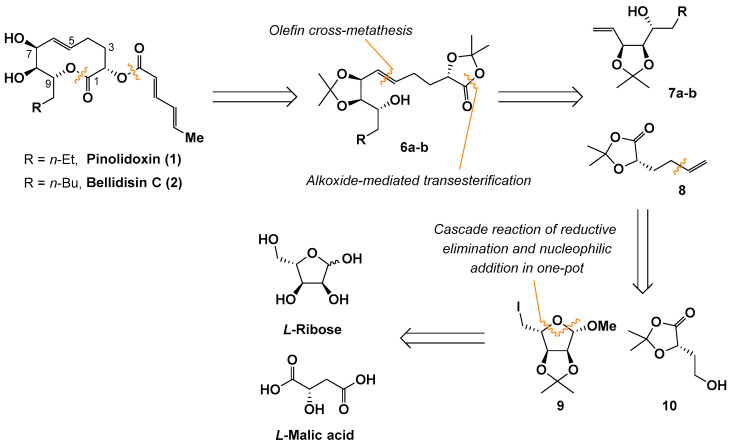
Retrosynthetic analysis of pinolidoxin and bellidisin C.

The retrosynthetic strategy for pinolidoxin (**1**) and bellidisin C (**2**) was depicted in Scheme 1. Initially, we envisaged that decanolides **1** and **2** could be elaborated from dioxolanones **6a**/**b** by sodium-hydride-promoted one-pot intramolecular lactonization, followed by esterification with sorbic acid and deprotection. The *trans* double bond at C5-C6 in **6a**/**b** could be installed by coupling triols **7a**/**b** with the olefinic side chain **8** through the olefin cross-metathesis (CM) strategy. The masked triols **7a**/**b**, in which three consecutive stereocenters are in place, could be accessible from the iodide **9** through a cascade reaction of reductive elimination and nucleophilic addition in a one-pot process as previously developed by us [26,27]. The primary iodide **9** would be readily accessible, obtained from commercially available *L*-ribose, whereas the alcohol **10** could be derived from *L*-malic acid through iodination and two-carbon homologation. Details of the studies thus undertaken are described below.

**Scheme 2:**
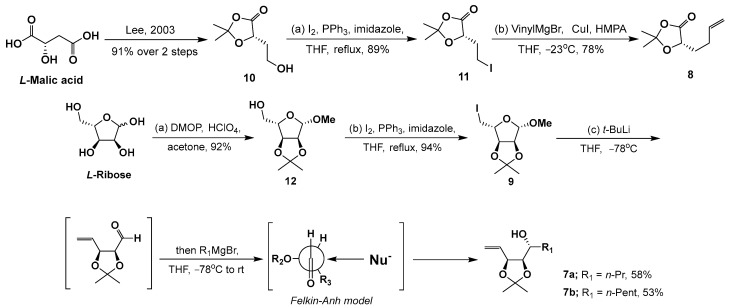
Synthesis of dioxolanone **8** [28] and triols **7a**,**b**.

Synthesis of the precursors dioxolanone **8** and triols **7a**,**b** were presented in Scheme 2. The known alcohol **10**, easily obtained from *L*-malic acid in 91% of overall yield in two steps according to a literature procedure [28], was subjected to a standard iodination condition to give iodide **11** in 89% yield. Subsequently, further CuI-catalyzed coupling of iodide **11** and vinylmagnesium bromide in the presence of HMPA [29] furnished terminal alkene **8** in a satisfactory yield (78%).

The synthesis of triols **7a** and **7b** began with conversion of commercially available *L*-ribose into the corresponding iodide **9** in two steps according to our reported procedure [30] (Scheme 2). The primary iodide **9** was treated sequentially with *t*-buthyllithium through a cascade reaction of a Bernet–Vasella-type reductive elimination and followed by a nucleophilic addition with *n*-propyl magnesium bromide to deliver the known alcohol **7a** [20] as a separable mixture of diastereomers (*anti*/*syn* = 2.7:1, 1H NMR analysis of the crude product) in 80% combined yield. Attempts to improve the ratio of *anti*/*syn* selectivity by changing solvents or reacting at lower temperatures were fruitless. Similarly, treatment of the iodide **9** with *t*-buthyllithium and *n*-pentyl magnesium bromide also afforded the desired *anti*-diastereomer **7b** as major diastereomer in a 75% combined yield (*anti*/*syn* = 2.4: 1) under the optimized conditions. It is noteworthy that this three-step in one-pot sequence involves an initial ring-opening by a lithium–iodide exchange and *β*-elimination, followed by a stereoselective nucleophilic addition to the intermediate hemiacetal [26,27]. The observed *anti*-stereoselectivity in the formation of **7a**/**b** would be explained by the steric hindrance of the bulky cyclic acetonide, which prevents the coordination of magnesium ion at the *α* site.

**Scheme 3:**
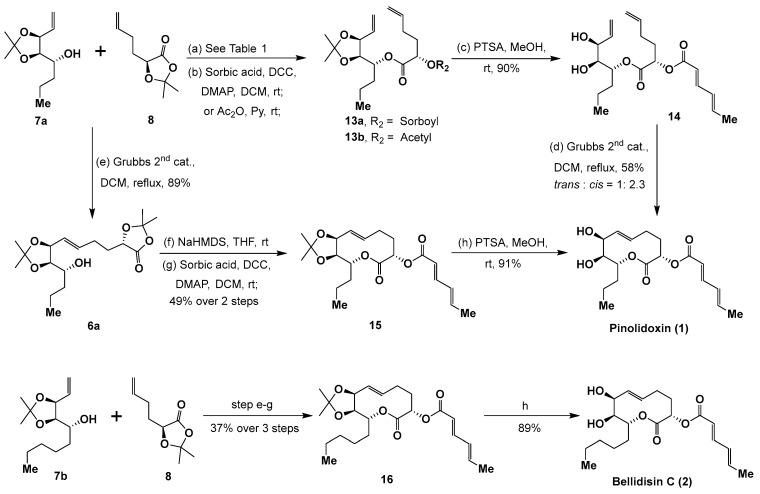
Synthesis of pinolidoxin (**1**) and bellidisin C (**2**).

With both key building blocks in hand, the stage was set for completion of the total synthesis of pinolidoxin (**1**) and bellidisin C (**2**), as summarized in Scheme 3. However, the envisaged assembly between triols **7a**/**b** and dioxolanone **8** turned out to be more difficult than we expected. On the basis of literature precedent [31,32,33] and our previous procedure [34,35], a variety of reaction conditions (reagents and solvents) were examined, and some of the representative results are shown in Table 1. Initially, treatment of the dioxolanones **8** with triol **7a** under De Brabander’s transesterification conditions (NaH in DMF or THF) [31] resulted in the recovery of starting material triol **7a** (entries 1–3) or led to unidentified decomposition products (entry 4). The acid-mediated esterification strategy [36] was also examined, and no desired ring-opening product was observed (entry 5). After screening several conditions, we were pleased to find that the alkoxide-mediated transesterification was most effective in the presence of NaHMDS [37] in anhydrous THF, leading to acetyl ester **13b** in a satisfactory yield (66%).

Esterification of the resulting secondary hydroxyl group **13** with commercially available sorbic acid was performed under a Keck esterification condition [38], affording the corresponding sorbate ester **13a** in a satisfactory yield of 61% over two steps. Hydrolysis of the isopropylidene group in **13a** with a *p*-toluenesulfonic acid (PTSA) in methanol furnished diol **14** in a high yield of 90%. Ring-closing metathesis (RCM) of **13a** using Grubbs second-generation catalyst [39] in refluxing CH_2_Cl_2_ predominantly afforded undesired *Z*-decanolide [19] in a 67% yield, with *Z*/*E* > 20:1. As a comparison, ring-closing metatheses of less-hindered diol **14** were carried out to deliver a separable mixture of pinolidoxin (**1**) and *Z*-isomer (*E*/*Z* = 1:2.3) in a 58% yield. Attempts to suppress the undesired *Z*-isomer by using benzoquinone or phenol as additives or running at higher temperature were fruitless. The low *E*/*Z* selectivity of the cross-metathesis reaction probably originated from the strong ring strain at the cycloalkene stage in the transition. Similar observations were also made by Kozmin and co-workers [19].

Although the synthesis of pinolidoxin (**1**) was short (eight longest linear steps) and efficient, it suffered from low *E*/*Z* selectivity and yield in the RCM process [40,41]. Moreover, in view of the similar structural skeleton of pinolidoxin (**1**) and bellidisin C (**2**), it is therefore highly desirable to develop a more efficient synthetic strategy for the total synthesis of both natural products. Accordingly, we revised our synthetic expedition towards pinolidoxin (**1**) and bellidisin C (**2**). Exposure of the dioxolanones **8** with triol **7a** in the presence of a Grubbs second-generation catalyst delivered *trans*-olefin **6a** in an excellent yield of 89% without any detectable *cis*-isomer product (Scheme 3). Subsequently, sodium–alkoxide-promoted intramolecular lactonization of **6a**, followed by esterification with sorbic acid, was carried out following a similar synthetic sequence to furnish the desired ten-membered lactone **15** with a satisfactory result over two steps. Interestingly, the NMR spectra of lactone **15** in CDCl_3_ solution at room temperature revealed the presence of two slowly interconverting conformers [42,43] in a ratio of about 4.3:1, which give rise to two sets of partly overlapped signals (see the Supplementary Materials). Similar observations for conformational equilibrium were also made by Fürstner [18] and Marco [20] in their synthesis of **1**, respectively. Finally, following the same acetonide deprotection, the synthesis of pinolidoxin (**1**) was accomplished in 91% yield.

Having developed an efficient route to the decanolide backbone, our focus was then shifted to the synthesis of bellidisin C (**2**). Accordingly, following the similar synthetic strategy as described above, assembly between triol **7b** and dioxolanones **8** was subjected to the cross-metathesis reaction and alkoxide-mediated intramolecular transesterification, followed by esterification with sorbic acid, successfully affording ten-membered lactone **16** in an overall yield of 37% for three steps. Finally, hydrolysis removal of the acetonide group in **16** was achieved with *p*-toluenesulfonic acid (PTSA) at room temperature, delivering bellidisin C (**2**) in an 89% yield. The spectroscopic data (^1^H and ^13^C NMR, HRMS)and optical rotation of synthetic pinolidoxin (**1**) and bellidisin C (**2**) were in excellent agreement with the literature reports [11,18,19].

## 3. Materials and Methods

### 3.1. General Experimental Procedures

All reagents were purchased from commercial corporations. All commercially available solvents were dried according to the standard procedures before use, and all reaction process was monitored by TLC (thin layer chromatography). Unless noted otherwise, commercially available materials were used without further purification. The crude products were purified by FC (flash chromatography) using 100–200 mesh silica gel. Infrared spectra were recorded using a FT-IR spectrophotometer with wave numbers expressed in cm^−1^. The optical rotation data were measured using a polarimeter at 25 °C. HRMS (high-resolution mass spectrometry) were recorded by FT-ICR-MS (Fourier-transform mass spectrometry), and the solvent was chromatographically pure CHCl_3_ or methanol. ^1^H NMR (400 MHz) and ^13^C NMR (100 MHz) were measured on Bruker topspin spectrometers (CDCl_3_ with TMS as an internal standard and coupling constants (*J*) in Hz.). Chemical shifts (*δ*) are given in ppm relative to residual solvent (usually CDCl_3_ or CD_3_OD, *δ* 7.26 (^1^H NMR) or 77.3 (^13^C NMR) for CDCl_3_; *δ* 3.31 (^1^H NMR) or 49.0 (^13^C NMR) for CD_3_OD for proton decoupled; Peak s means singlet state; d means doublet state; t means triplet state; q means quartet state; m means multiple state.

### 3.2. Experimental Procedures

#### 3.2.1. Synthesis of Compound **11**

The known compound **10** can be readily prepared from *L*-malic acid in 87% of the overall yield in two steps using the procedure reported by Lee [28]. To a solution of compound **10** (3.2 g, 20 mmol) in THF (80 mL) at 0 °C, imidazole (2.72 g, 40 mmol,) and PPh_3_ (7.8 g, 30 mmol) were added. After stirring at the same temperature for 10 min, solid I_2_ (7.6 g, 30 mmol) was added portion-wise. The mixture was heated to reflux for further 1.5 h. After completion of the reaction, the solution was cooled to room temperature and quenched with 20 mL saturated solution of Na_2_S_2_O_3_. The mixture was extracted with ethyl acetate (2 × 100 mL). The combined organic phase was dried over anhydrous Na_2_SO_4_ and concentrated in vacuum. Purification of the crude product by flash column chromatography (petroleum ether/EtOAc 8:1) gave compound **11** (4.81 g, 89%) as a colorless oil. ${\left[\alpha \right]}_{D}^{25}=$ +23.4 (*c* 0.1, CHCl_3_); ^1^H NMR (400 MHz, Chloroform-*d*): *δ* 4.47 (dd, *J* = 8.0, 4.4 Hz, 1H), 3.34–3.20 (m, 2H), 2.38 (ddt, *J* = 15.2, 4.4, 7.6 Hz, 1H), 2.17 (ddt, *J* = 15.2, 5.4, 7.6 Hz, 1H), 1.60 (s, 3H), 1.55 (s, 3H); ^13^C NMR (100 MHz, Chloroform-*d*): *δ* 172.9, 111.6, 74.42, 74.40, 36.1, 27.8, 26.4. HRMS (ESI): calcd. For C_7_H_11_IO_3_Na^+^[M + Na]^+^, 292.9645; found 292.9645.

#### 3.2.2. Synthesis of Compound **8**

To a solution of compound **11** (1.074g, 3.98 mmol) in anhydrous THF (30 mL) were added CuI (75.8 mg, 0.4 mmol), vinylmagnesium bromide (11.18 mL, 1.0 M in THF, 11.18 mmol), and hexamethylphosphoric triamide (HMPA, 4.09 mL, 24 mmol) at −78 °C under a nitrogen atmosphere. The reaction was warmed to room temperature within 2 h. The mixture was stirred at the same temperature for 3 h. After completion of the reaction, the mixture was quenched with saturated aqueous NH_4_Cl (10 mL) at room temperature. The mixture was extracted with EtOAc (60 mL). The organic phase was dried over anhydrous Na_2_SO_4_ and concentrated in vacuum. Purification of the crude product by flash column chromatography (petroleum ether/EtOAc 10:1) gave the compound **8** (528 mg, 78%) as a colorless oil. ${\left[\alpha \right]}_{D}^{25}$ = +122.2 (*c* 0.1, CHCl_3_); ^1^H NMR (400 MHz, Chloroform-*d*): *δ* 5.78 (ddt, *J* = 16.8, 10.2, 6.6 Hz, 1H), 5.27–4.80 (m, 2H), 4.38 (dd, *J* = 7.2, 4.4 Hz, 1H), 2.22–2.17 (m, 2H), 2.00–1.87 (m, 1H), 1.86–1.72 (m, 1H), 1.58 (s, 3H), 1.51 (s, 3H); ^13^C NMR (100 MHz, Chloroform-d): *δ* 173.2, 136.8, 115.8, 110.4, 73.3, 30.7, 28.9, 27.1, 25.7. HRMS (ESI): calcd. For C_9_H_14_O_3_Na^+^[M + Na]^+^, 193.0835; found 193.0836.

#### 3.2.3. Synthesis of Compound **7a**

The known iodide **9** was easily obtained from commercially available *L*-ribose in two steps according to our reported procedure [30]. To a solution of compound **9** (2.07 g, 6.6 mmol) in anhydrous THF (50 mL) was added *t*-BuLi (12.6 mL, 1.3 M in Pentane, 16.4 mmol) at −78 °C under a nitrogen atmosphere. The mixture was stirred at same temperature for 30 min, and *n*-PrMgBr was added (13.2 mL, 2 M in THF, 26.4 mmol). The mixture was stirred at −78 °C for 2 h. After completion of the reaction, the solution was warmed to room temperature and quenched with saturated 40 mL solution of NH_4_Cl. The mixture was extracted with ethyl acetate (2 × 50 mL). The organic phase was dried over anhydrous Na_2_SO_4_ and concentrated in vacuum. Purification of the crude product by flash column chromatography (petroleum ether/EtOAc 10:1) gave known compound **7a** (765 mg, 58%) as colorless oil and minor diastereomer *cis*-**7a** (283 mg, 22%) as colorless oil. For compound **7a**: ${\left[\alpha \right]}_{D}^{25}=$ +40.4 (*c* 0.1, CHCl_3_); ^1^H NMR (400 MHz, Chloroform-*d*): *δ* 6.04 (ddd, *J* = 17.6, 10.2, 7.6 Hz, 1H), 5.42 (dd, *J* = 17.2, 1.2 Hz, 1H), 5.31 (dd, *J* = 10.2, 1.2 Hz, 1H), 4.68–4.60 (m, 1H), 3.97 (dd, *J* = 8.2, 6.4 Hz, 1H), 3.67 (td, *J* = 8.4, 2.8 Hz, 1H), 1.68 (tq, *J* = 7.2, 2.8 Hz, 2H), 1.63–1.49 (m, 1H), 1.48 (s, 3H), 1.46–1.40 (m, 2H), 1.37 (s, 3H), 0.94 (t, *J* = 7.2 Hz, 3H); ^13^C NMR (100 MHz, Chloroform-*d*): *δ* 134.8, 118.6, 108.7, 80.8, 79.0, 69.8, 35.9, 27.8, 25.3, 18.4, 14.1. IR (film): 3449.1, 2963.6, 2932.4, 2871.7, 1455.5, 1379.6, 1370.8, 1214.6, 1167.6, 1099.2, 1015.8, 924.9, 872.1, 512.0 cm^−1^. HRMS (ESI): calcd. For C_11_H_20_O_3_Na^+^[M + Na]^+^, 223.1305; found 223.1304. The spectra data of **7a** were identical to those reported [20].

For *cis-***7a**: ${\left[\alpha \right]}_{D}^{25}=$−121.3 (c 0.1, CHCl_3_); ^1^H NMR (400 MHz, Chloroform-*d*): *δ* 6.05 (ddd, *J* = 17.2, 10.3, 8.2 Hz, 1H), 5.37 (dt, *J* = 17.2, 1.4 Hz, 1H), 5.29 (dd, *J* = 10.3, 1.8 Hz, 1H), 4.57 (dd, *J* = 8.2, 6.0 Hz, 1H), 4.14 (dd, *J* = 9.4, 5.9 Hz, 1H), 3.31 (d, *J* = 9.3 Hz, 1H), 1.70 (s, 1H), 1.47 (s, 3H), 1.40 (d, *J* = 6.5 Hz, 1H), 1.35 (s, 3H), 1.05–1.25 (m, 1H), 0.98 (s, 4H), 0.96–0.82 (m, 1H); ^13^C NMR (100 MHz, Chloroform-*d*): *δ* 135.8, 118.5, 109.1, 80.3, 78.2, 75.9, 34.8, 28.2, 26.1, 26.1, 25.6. HRMS (ESI): calcd. For C_11_H_20_O_3_Na^+^[M + Na]^+^, 223.1305; found 223.1306.

#### 3.2.4. Synthesis of Compound **7b**

To a solution of compound **9** (500 mg, 1.59 mmol) in anhydrous THF (10 mL) was added *t*-BuLi (3 mL, 1.3 M in Pentane, 3.9 mmol) at −78 °C under a nitrogen atmosphere. The mixture was stirred at the same temperature for 30 min, and *n*-PentMgBr was added (3 mL, 2 M in THF, 6.372 mmol). The mixture was stirred at the same temperature for 2 h. After completion of the reaction, the mixture was quenched with saturated aqueous NH_4_Cl (5 mL) at room temperature. The mixture was extracted with EtOAc (40 mL). The organic phase was dried over anhydrous Na_2_SO_4_ and concentrated in vacuum. Purification of the crude product by flash column chromatography (petroleum ether/EtOAc 10:1) gave the compound **7b** (192 mg, 53%) and minor diastereomer *cis*-**7b** (80 mg, 22%) as colorless oil, respectively. ${\left[\alpha \right]}_{D}^{25}=$ +26.3 (*c* 0.1, CHCl_3_); ^1^H NMR (400 MHz, Chloroform-*d*): *δ* 6.03 (ddd, *J* = 17.2, 10.2, 7.6 Hz, 1H), 5.41 (dt, *J* = 17.2, 1.4 Hz, 1H), 5.30 (dt, *J* = 10.2, 1.4 Hz, 1H), 4.67–4.59 (m, 1H), 3.96 (dd, *J* = 8.2, 6.4 Hz, 1H), 3.65 (td, J = 8.4, 2.8 Hz, 1H), 1.82–1.59 (m, 2H), 1.56–1.49 (m, 1H), 1.47 (s, 3H), 1.43–1.38 (m, 1H), 1.36 (s, 3H), 1.34–1.30 (m, 4H), 0.93–0.82 (m, 4H); ^13^C NMR (100 MHz, Chloroform-*d*): *δ* 134.8, 118.5, 108.7, 80.7, 79.0, 70.0, 33.7, 31.8, 27.8, 25.3, 24.9, 22.7, 14.1. IR (film): 3427.2, 2954.6, 2932.8, 2865.3, 1461.4, 1379.3, 1369.4, 1240.9, 1215.4, 1167.5, 1056.9, 1038.7, 972.6, 924.5, 872.99, 512.3 cm^−1^. HRMS (ESI): calcd. For C_13_H_24_O_3_Na^+^[M + Na]^+^, 251.1618; found 251.1618.

For *cis*-**7b**: ${\left[\alpha \right]}_{D}^{25}=$ 103.8 (*c* 0.1, CHCl_3_); ^1^H NMR (400 MHz, Chloroform-*d*): *δ* 5.97 (ddd, J = 17.2, 10.2, 8.2 Hz, 1H), 5.38–5.29 (m, 2H), 4.56 (dd, *J* = 8.2, 6.8 Hz, 1H), 4.01 (dd, *J* = 6.8, 5.4 Hz, 1H), 3.55 (dt, *J* = 6.8, 5.4 Hz, 1H), 2.05 (*br* s, 1H), 1.51 (s, 3H), 1.42 (d, *J* = 7.2 Hz, 3H), 1.38 (s, 3H), 1.32–1.26 (m, 5H), 0.88 (t, *J* = 6.8, 3H); ^13^C NMR (100 MHz, Chloroform-*d*): *δ* 134.1, 119.3, 108.6, 80.8, 79.1, 69.7, 33.8, 31.7, 27.5, 25.2, 25.1, 22.6, 14.0. HRMS (ESI): calcd. For C_13_H_24_O_3_Na^+^[M + Na]^+^, 251.1618; found 251.1618.

#### 3.2.5. Synthesis of Compounds **13a** and **13b**

To a solution of compound **7a** (120 mg, 0.6 mmol) in THF (15 mL) at room temperature was added NaHMDS (0.6 mL, 2.0 M in THF, 1.2 mmol) under a nitrogen atmosphere. The mixture was stirred at the same temperature for 15 min, and **8** was added (152 mg in 3 mL THF, 0.9 mmol). The mixture was stirred at the same temperature for 5 min. After completion of the reaction, the mixture was quenched with saturated aqueous NH_4_Cl (3 mL) at room temperature. The mixture was extracted with EtOAc (20 mL). The organic phase was dried over anhydrous Na_2_SO_4_ and concentrated in vacuum to afford crude compound **13**. The ester **13** was unstable and decomposed rapidly on a flash column or standing in air. Thus, the ester **13** was converted into the corresponding acetyl ester **13a** or **13b** under standard acetylation conditions or Keck esterification condition.

For compound **13a**: To a solution of the crude **13** in anhydrous DCM (15 mL) were added sorbic acid (200 mg, 1.8 mmol), DCC (491 mg, 2.4 mmol), and DMAP (36 mg, 0.3 mmol). The mixture was stirred at room temperature for 13 h and quenched with MeOH (3 mL). The mixture was concentrated under reduced pressure and purified via flash chromatography on silica gel (petroleum ether/EtOAc 20:1) to afford **13a** (149 mg, 61% over two steps) as a colorless oil. ^1^H NMR (400 MHz, Chloroform-*d*): *δ* 7.31–7.24 (m, 1H), 6.24–6.18 (m, 2H), 5.87–5.70 (m, 3H), 5.36 (d, *J* = 17.2 Hz, 1H), 5.25 (d, *J* = 10.4 Hz, 1H), 5.04–4.93 (m, 4H), 4.62 (d, *J* = 6.8 Hz, 1H), 4.18 (t, *J* = 6.8 Hz, 1H), 2.17 (d, *J* = 7.4 Hz, 2H), 1.92 (d, *J* = 8.0 Hz, 2H), 1.85 (d, *J* = 5.2 Hz, 3H), 1.68–1.58 (m, 2H), 1.46 (s, 3H), 1.35 (s, 3H), 1.28–1.17 (m, 2H), 0.88 (t, *J* = 7.2 Hz, 3H); ^13^C NMR (100 MHz, Chloroform-*d*): *δ* 169.5, 166.4, 146.2, 140.2, 136.7, 132.6, 129.7, 119.1, 117.8, 115.9, 108.8, 78.7, 78.0, 72.8, 71.3, 33.0, 30.4, 29.4, 27.5, 25.3, 18.7, 17.7, 14.0. HRMS (ESI): calcd. For C_23_H_34_O_6_Na^+^[M + Na]^+^, 429.2248; found 429.2250.

For compound **13b**: The above transesterifications were carried out with **7a** (40 mg, 0.2 mmol) and **8** (17 mg, 0.1 mmol). To a solution of the crude **13** in anhydrous pyridine (5 mL) was added acetic anhydride (0.165 mL, 1.8 mmol) at room temperature. The mixture was stirred at the same temperature for another 1 h and quenched with methanol (3 mL). The mixture was concentrated under vacuum. The resulting residue was purified using flash chromatography on silica gel (petroleum ether/EtOAc 8:1) to afford **13b** (24 mg, 66% over two steps) as a colorless oil. ^1^H NMR (400 MHz, Chloroform-*d*): *δ* 5.84–5.79 (m, 2H), 5.38 (d, *J* = 16.8 Hz, 1H), 5.27 (d, *J* = 10.4 Hz, 1H), 5.08–4.96 (m, 4H), 4.63 (t, *J* = 6.8 Hz, 1H), 4.19 (t, *J* = 6.8 Hz, 1H), 2.24–2.15 (m, 2H), 2.13 (s, 3H), 1.95–1.82 (m, 2H), 1.65 (q, *J* = 7.2 Hz, 2H), 1.48 (s, 3H), 1.37 (s, 3H), 1.34–1.21 (m, 2H), 0.90 (t, *J* = 7.2 Hz, 3H); ^13^C NMR (100 MHz, Chloroform-d): *δ* 170.3, 169.3, 136.6, 132.5, 119.1, 116.0, 108.8, 78.7, 78.0, 73.0, 71.5, 33.0, 30.2, 29.4, 27.5, 25.3, 20.6, 17.8, 14.0. HRMS (ESI): calcd. For C_21_H_34_O_6_Na^+^[M + Na]^+^, 377.1935; found 377.1936.

#### 3.2.6. Synthesis of Compound **14**

To a solution of the crude compound **13a** (28 mg, 0.069 mmol) in MeOH (8 mL) was added PTSA (2.3 mg, 0.014mmol). The mixture was stirred at room temperature for 10 h and quenched with Et_3_N (0.3 mL). The mixture was concentrated under reduced pressure and purified via flash chromatography on silica gel (petroleum ether/EtOAc 1:1) to afford **14** (23 mg, 90%) as a colorless oil. ^1^H NMR (400 MHz, Chloroform-*d*): *δ* 7.28–7.25 (m, 1H), 6.21–6.18 (m, 2H), 6.04–5.90 (m, 1H), 5.89–5.73 (m, 2H), 5.52 (dd, *J* = 6.8, 4.0 Hz, 1H), 5.37 (m, 1H), 5.34 (d, *J* = 10.6 Hz, 1H), 5.14–4.93 (m, 3H), 3.73 (dd, *J* = 6.4, 4.0 Hz, 1H), 3.63–3.54 (m, 1H), 2.22–2.17 (m, 4H), 2.05–1.95 (m, 2H), 1.90–1.84 (m, 3H), 1.73–1.63 (m, 2H), 1.41–1.25 (m, 2H), 0.93 (t, *J* = 7.2 Hz, 3H); ^13^C NMR (100 MHz, Chloroform-*d*): *δ* 170.3, 166.9, 146.6, 140.6, 136.6, 136.0, 129.7, 117.9, 117.6, 116.0, 75.4, 74.2, 73.2, 71.9, 31.7, 30.3, 29.3, 18.8, 18.3, 13.9. HRMS (ESI): calcd. For C_20_H_30_O_6_Na^+^[M + Na]^+^, 389.1935; found 389.1940.

#### 3.2.7. Synthesis of Pinolidoxin (1) and **14a** (Z Isomer) from Compound **14**

To a stirred solution of **14** (30 mg, 0.082 mmol) in anhydrous DCM (20 mL) was added the Grubbs second-generation catalyst (4.25 mg, 0.05 mmol). The mixture was refluxed for 2 h and concentrated under reduced pressure. Purification of the crude product using flash chromatography on silica gel (petroleum ether/EtOAc 2:1) afforded pinolidoxin (5 mg, 17.5%) as a colorless oil and compound **14a** (Z isomer of pinolidoxin) (12 mg, 40%) as a colorless oil.

For pinolidoxin (**1**): ${\left[\alpha \right]}_{D}^{25}$ = +133.6 (c 0.2, MeOH) {Lit.^12^ ${\left[\alpha \right]}_{D}^{25}$ = +142.9 (c 0.31, MeOH)}; ^1^H NMR (400 MHz, Chloroform-*d*): *δ* 7.31 (dd, *J* = 15.4, 9.6 Hz, 1H), 6.24–6.21 (m, 2H), 5.88 (d, *J* = 15.4 Hz, 1H), 5.65 (dd, *J* = 15.6, 1.4 Hz, 1H), 5.53 (td, *J* = 15.6, 7.4 Hz, 1H), 5.26 (dd, *J* = 5.8, 1.8 Hz, 1H), 5.04 (td, *J* = 9.4, 2.8 Hz, 1H), 4.44 (*br* s, 1H), 3.52 (dd, *J* = 10.0, 2.4 Hz, 1H), 2.43–2.40 (m, 1H), 2.26–2.16 (m, 2H), 2.08–2.00 (m, 1H), 1.89 (d, *J* = 5.6 Hz, 3H), 1.88–1.78 (m, 1H), 1.53–1.49 (m, 1H), 1.37–1.31 (m, 1H), 1.25–1.21 (m, 1H), 0.87 (t, *J* = 7.2 Hz, 3H); ^13^C NMR (100 MHz, Chloroform-d): *δ* 171.9, 166.0, 145.9, 140.3, 132.3, 129.7, 123.0, 118.1, 73.2, 73.1, 71.3, 69.7, 33.6, 29.8, 27.4, 18.7, 17.4, 13.8. IR (film): 3473.9, 2960.3, 2927.6, 2871.6, 1714.6, 1643.5, 1297.4, 1242.1, 1182.4, 1133.9, 1100.6, 1076.3, 1026.9, 997.8, 955.3, 866.7, 735.1, 698.5, 501.5 cm^−1^. HRMS (ESI): calcd. For C_18_H_26_O_6_Na^+^[M + Na]^+^, 361.1622; found 361.1622;

For compound **14a** (Z isomer of pinolidoxin): ^1^H NMR (400 MHz, Chloroform-*d*): *δ* 7.37–7.25 (m, 1H), 6.22 (dd, *J* = 6.8, 3.2 Hz, 2H), 5.84 (d, *J* = 15.4 Hz, 1H), 5.61–5.54 (m, 2H), 5.03 (t, *J* = 3.6 Hz, 1H), 4.76–4.59 (m, 2H), 3.92 (dd, *J* = 9.6, 4.0 Hz, 1H), 2.77 (d, *J* = 11.8 Hz, 1H), 2.55 (s, 1H), 2.18- 2.10 (m, 1H), 2.08–2.00 (m, 2H), 1.97–1.86 (m, 3H), 1.80–1.72 (m, 1H), 1.65–1.51 (m, 2H), 1.42–1.23 (m, 2H), 0.89 (t, *J* = 7.2 Hz, 3H); ^13^C NMR (100 MHz, Chloroform-*d*): *δ* 169.4, 166.4, 146.0, 140.4, 133.8, 129.6, 126.8, 117.8, 74.9, 73.9, 71.1, 66.7, 35.2, 30.5, 21.8, 18.7, 17.7, 14.0. IR (film): 3431.9, 2958.7, 2925.4, 1716.2, 1645.2, 1265.2, 1175.2, 1135.3, 1026.8, 999.5, 735.1 cm^−1^. HRMS (ESI): calcd. For C_18_H_26_O_6_Na^+^[M + Na]^+^, 361.1622; found 361.1623.

#### 3.2.8. Synthesis of Compound **6a**

To a stirred solution of **8** (200 mg, 1.18 mmol) and compound **7a** (470 mg, 2.35 mmol) in anhydrous DCM (30 mL) was added Grubbs second-generation catalyst (49.9 mg, 0.05 mmol). The mixture was refluxed for 8 h and concentrated under reduced pressure. Purification of the crude product via flash chromatography on silica gel (petroleum ether/EtOAc 5:1) afforded compound **6a** (358 mg, 89%) as a colorless oil. ${\left[\alpha \right]}_{D}^{25}$ = +45.7 (*c* 0.1, CHCl_3_); ^1^H NMR (400 MHz, Chloroform-d): *δ* 5.82 (dd, *J* = 15.2, 6.4 Hz, 1H), 5.72 (dd, *J* = 15.2, 8.0 Hz, 1H), 4.61 (t, *J* = 7.2 Hz, 1H), 4.40 (dd, *J* = 7.6, 4.2 Hz, 1H), 3.92 (dd, *J* = 8.0, 6.4 Hz, 1H), 3.65 (td, *J* = 8.4, 2.8 Hz, 1H), 2.00 (ddt, *J* = 15.4, 4.4, 7.6 Hz, 2H), 1.84 (dq, *J* = 14.6, 7.6 Hz, 1H), 1.73–1.62 (m, 3H), 1.60 (s, 3H), 1.53 (s, 3H), 1.46 (s, 3H), 1.38 (m, 3H), 1.35 (s, 3H), 0.94 (t, *J* = 7.0 Hz, 3H); ^13^C NMR (100 MHz, Chloroform-*d*): *δ* 173.1, 133.4, 127.8, 110.6, 108.5, 80.7, 78.6, 73.3, 69.8, 35.9, 30.9, 27.9, 27.7, 27.2, 25.7, 25.3, 18.4, 14.1. IR (film): 3458.3, 2960.3, 2932.3, 1874.7, 1778.9, 1379.8, 1214.6, 1167.4, 1097.1, 1065.5, 1031.3, 926.6, 871.8, 736.5, 702.9, 511.8 cm^−1^. HRMS (ESI): calcd. For C_18_H_30_O_6_Na^+^[M + Na]^+^, 365.1935; found 365.1935.

#### 3.2.9. Synthesis of Compound **15**

To a solution of compound **6a** (104 mg, 0.3 mmol) in THF (15 mL) at room temperature under a nitrogen atmosphere was added NaHMDS (0.3 mL, 2.0 M in THF, 0.6 mmol). The mixture was stirred at room temperature for 20 min. After completion of the reaction, the mixture was quenched with saturated aqueous NH_4_Cl (3 mL) at room temperature. The mixture was extracted with EtOAc (30 mL). Then, the solution mixture was evaporated under a reduced pressure to afford the crude lactone **15a**. The lactone **15a** was unstable and decomposed rapidly on the flash column or standing in air. Thus, **15a** was converted into the corresponding sorbate **15** under the standard Keck esterification condition. To a solution of the crude compound **15a** in anhydrous DCM (15 mL) were added DMAP (8.6 mg, 0.07 mmol), DCC (184 mg, 0.95 mmol), and sorbic acid (79 mg, 0.71 mmol) at room temperature. The mixture was stirred at the same temperature for another 16 h and quenched with 3 mL methanol. The mixture was concentrated under vacuum. The residue was purified using flash chromatography on silica gel (petroleum ether/EtOAc 30:1) to afford compound **15** (56 mg, 49% over 2 steps) as a colorless oil. ${\left[\alpha \right]}_{D}^{25}$ = +163.3 (*c* 0.1, CHCl_3_); *The NMR spectra of **15** show the presence of two conformers in a ratio of 4.3:1, which give rise to two sets of partly overlapped signals (the minor conformers are given in italics).* ^1^H NMR of two conformers (400 MHz, Chloroform-*d*): *δ* 7.33 (dd, *J* = 15.4, 9.2 Hz, 1.23H), 6.31–6.17 (m, 2.5H), 5.92–5.76 (m, 2H), 5.67 (ddd, *J* = 15.6, 10.2, 3.2 Hz, 1.23H), *5.55 (d, J = 8.2 Hz, 0.23H)*, 5.29 (d, *J* = 6.0 Hz, 1H), *5.15 (t, J = 6.0 Hz, 0.23H)*, 4.95 (t, *J* = 9.0 Hz, 1H), *4.81 (s, 0.23H)*, 4.65 (s, 1H), *4.72 (t, J = 7.4 Hz, 0.23H)*, *4.07 (dd, J = 10.0, 6.7 Hz, 0.23H)*, 3.93 (dd, *J* = 10.0, 4.6 Hz, 1H), 2.48–2.32 (m, 2.5H), 2.25 (td, *J* = 15.6, 11.4 Hz, 2.5H), 2.03 (dd, *J* = 11.4, 6.0 Hz, 1.23H), 1.89 (d, *J* = 5.6 Hz, 3.7H), 1.72 (t, *J* = 7.2 Hz, 1.23H), 1.53 (s, 3.7H), 1.36 (s, 3.7H), 0.89 (t, *J* = 7.2 Hz, 3.7H); ^13^C NMR of major conformer (100 MHz, Chloroform-*d*): *δ* 172.2, 166.0, 145.9, 140.3, 129.7, 128.8, 123.0, 118.2, 109.2, 77.9, 76.1, 71.7, 70.1, 34.0, 30.6, 28.5, 27.7, 26.2, 18.8, 17.6, 13.9. HRMS (ESI): calcd. For C_21_H_30_O_6_Na^+^[M + Na]^+^, 401.1935 found: 401.1934.

#### 3.2.10. Synthesis of Pinolidoxin (1) from Compound **15**

To a solution of compound **15** (15 mg, 0.04 mmol) in MeOH (5 mL) was added PTSA (2 mg, 0.009 mmol). The mixture was stirred at room temperature for 10 h and quenched with Et_3_N (0.3 mL). The mixture was concentrated under reduced pressure. Purification of the crude product using flash chromatography on silica gel (petroleum ether/EtOAc 1:1) afforded pinolidoxin **(1)** (12 mg, 91%) as a colorless oil.

#### 3.2.11. Synthesis of Compound **6b**

Compounds **7b** and **8** were subjected to the same cross-metathesis to furnish **6b** in a yield of 81%. ${\left[\alpha \right]}_{D}^{25}=$ +127.4 (*c* 0.1, CHCl_3_); ^1^H NMR (400 MHz, Chloroform-d): *δ* 5.90–5.77 (m, 1H), 5.77–5.66 (m, 1H), 4.61 (dd, *J* = 8.0, 6.4 Hz, 1H), 4.40 (dd, *J* = 7.6, 4.4 Hz, 1H), 3.92 (dd, *J* = 8.2, 6.4 Hz, 1H), 3.63 (td, *J* = 8.4, 2.8 Hz, 1H), 2.52 (*br* s, 1H), 2.37–2.19 (m, 2H), 2.06–1.93 (m, 1H), 1.84 (dtd, *J* = 14.2, 8.0, 6.4 Hz, 1H), 1.70 (ddt, *J* = 15.2, 13.2, 2.8 Hz, 2H), 1.61 (s, 3H), 1.54 (s, 3H), 1.46 (s, 3H), 1.45–1.37 (m, 1H), 1.35 (s, 3H), 1.29 (s, 5H), 0.95 (t, *J* = 7.0 Hz, 3H); ^13^C NMR (100 MHz, Chloroform-*d*): *δ* 173.2, 133.5, 127.8, 110.7, 108.5, 80.7, 78.6, 73.3, 70.1, 33.7, 31.9, 30.9, 27.9, 27.7, 27.2, 25.8, 25.3, 24.9, 22.7, 14.1. HRMS (ESI): calcd. For C_20_H_34_O_6_Na^+^[M + Na]^+^, 393.2248; found 393.2248.

#### 3.2.12. Synthesis of Compound **16**

Compound **6b** was subjected to the same transesterification followed by esterification to afford lactone **16** in a yield of 46% over two steps. *The NMR spectra of **16** also show the presence of two conformers in a ratio of 3.7:1, which give rise to two sets of partly overlapped signals (the minor conformers are given in italics).*$\;{\left[\alpha \right]}_{D}^{25}=$ +151.6 (*c* 0.1, CHCl_3_); ^1^H NMR of two conformers (400 MHz, Chloroform-*d*): *δ* 7.30 (dd, *J* = 15.2, 9.6 Hz, 1.27H), 6.31–6.13 (m, 2.5H), 5.92–5.61 (m, 2.27H), 5.64 (dd, J = 7.8, 2.6 Hz, 1.27H), *5.53 (dd, J = 16.0, 8.2 Hz, 0.27H)*, 5.29 (dd, *J* = 5.8, 1.6 Hz, 1H), *5.15 (dd, J = 6.8, 4.8 Hz, 0.27H)*, 4.94 (ddd, *J* = 10.8, 8.4, 3.0 Hz, 1H), *4.82 (s, 0.27H)*, *4.75–4.69 (m, 0.27H)*, 4.65 (dt, *J* = 5.4, 2.4 Hz, 1H), *4.07 (dd, J = 10.0, 6.6 Hz, 0.27H)*, 3.93 (dd, *J* = 10.0, 4.7 Hz, 1H), 2.48–2.32 (m, 2.5H), 2.32–2.18 (m, 3H), 2.04 (dt, *J* = 11.0, 3.0 Hz, 3.8H), 1.89 (d, *J* = 5.6 Hz, 3.8H), 1.73 (d, *J* = 13.4 Hz, 2.5H), 1.53 (s, 3.8H), 1.37 (s, 3.8H), 1.26 (d, *J* = 7.9 Hz, 5H), 0.85 (s, 3.8H); ^13^C NMR of major conformer (100 MHz, Chloroform-*d*): *δ* 172.2, 166.0, 145.9, 140.3, 129.7, 128.8, 123.0, 118.2, 109.2, 77.5, 76.1, 71.9, 70.1, 31.7, 31.5, 30.6, 28.5, 27.7, 26.3, 23.9, 22.5, 18.8, 14.0. HRMS (ESI): calcd. For C_23_H_34_O_6_Na^+^[M + Na]^+^, 429.2248 found 429.2241.

#### 3.2.13. Synthesis of Bellidisin C (2)

To a solution of compound **16** (17 mg, 0.042 mmol) in MeOH (5 mL) was added PTSA (2 mg, 0.009 mmol). The mixture was stirred at room temperature for 12 h and quenched with Et_3_N (0.3 mL). The mixture was concentrated under reduced pressure and purified via flash chromatography on silica gel (petroleum ether/EtOAc 1:1) to afford bellidisin C (13.6 mg, 89%) as a colorless oil. ${\left[\alpha \right]}_{D}^{25}=$ +121.9 (*c* 0.2, MeOH); {Lit.^11^ ${\left[\alpha \right]}_{D}^{25}$ = +138.14 (*c* 0.04, MeOH)}; ^1^H NMR (400 MHz, Chloroform-*d*): *δ* 7.31 (dd, *J* = 15.4, 10.0 Hz, 1H), 6.25–6.20 (m, 2H), 5.87 (d, *J* = 15.4 Hz, 1H), 5.66 (dd, *J* = 15.6, 2.0 Hz, 1H), 5.54–5.52 (m, 1H), 5.26 (dd, *J* = 5.4, 2.0 Hz, 1H), 5.00 (td, *J* = 10.0, 2.8 Hz, 1H), 4.44 (*br* s, 1H), 3.52 (dd, *J* = 10.0, 2.6 Hz, 1H), 2.44–2.39 (m, 1H), 2.26–2.18 (m, 2H), 2.03–1.99 (m, 1H), 1.89 (d, *J* = 6.0 Hz, 3H), 1.82–1.78 (m, 1H), 1.52–1.48 (m, 1H), 1.38–1.22 (m, 8H), 0.85 (t, *J* = 6.4 Hz, 3H); ^13^C NMR (100 MHz, Chloroform-*d*): *δ* 172.0, 166.0, 145.9, 140.3, 132.3, 129.7, 123.1, 118.1, 73.1 (d), 71.5, 69.7, 31.5, 31.4, 29.9, 27.4, 23.7, 22.4, 18.8, 14.0. IR (film): 3408.5, 2927.8, 2857.5, 1716.7, 1645.0, 1258.3, 1242.6, 1134.9, 998.1cm^−1^. HRMS (ESI): calcd. For C_20_H_30_O_6_Na^+^[M + Na]^+^ 389.1935; found 389.1936.

## 4. Conclusions

In conclusion, an enantioselective total synthesis of pinolidoxin and bellidisin C has been accomplished from readily available *L*-ribose and *L*-malic acid in the longest linear sequence of eight steps. In particular, bellidisin C, which is the first total synthesis so far, was achieved in eight longest linear sequences (19.1% overall yield). The synthetic route highlights the efficiency of our chiron-based approach to access the decanolide skeleton through a sodium-alkoxide-promoted intramolecular lactonization and a cascade reaction of reductive elimination and nucleophilic addition in a one-pot process. Further bioactivity evaluation and the synthesis of other decanolide-containing natural products, such as herbarumin and bellidisins B and D, are currently being explored in our laboratory.

## Figures and Tables

**Table 1:** Transesterification of dioxolanone **8** with **7a** under various conditions.

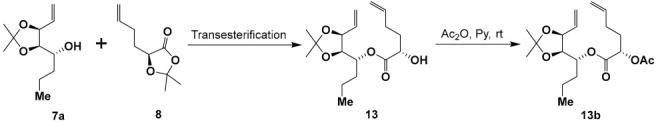				
Entry	Reagents and Conditions ^a^	Temperature	Solvent	Yieid of 13b (%) ^b^
1	NaH (1.5–6.0 eq)	−78 °C	THF	NR ^c^
2	NaH (2.0 eq)	0 °C	THF	Decomposition ^c,d^
3	NaH (2.0 eq)	0 °C to rt	THF	Decomposition ^c,d^
4	NaH (2.0 eq)	0° to rt	DMF	Decomposition ^d^
5	PTSA (0.1 eq)	rt to reflux	Toluene	Decomposition ^d,e^
6	NaHMDS (1.5 eq)	0 °C	THF	20%
7	NaHMDS (2.0 eq)	rt	THF	66%

^a^ Unless otherwise noted, the conditions were carried out with 0.2 mmol **7a** and 0.1 mmol **8**. ^b^ The ester **13** was unstable and decomposed rapidly on flash column or standing in air. Thus, the ester **13** was converted into the corresponding acetyl ester **13b** under standard acetylation conditions. ^c^ Starting material **7a** was recovered with unidentified mixtures. ^d^ With considerable unidentified products. ^e^ No desired product was observed.

## Data Availability

Data are contained within the article and Supplementary Materials.

## Supplementary Materials

The following supplementary materials can be downloaded at https://www.mdpi.com/article/10.3390/molecules29235500/s1; File S1: Copies of NMR spectra and IR Spectra and comparison of ^1^H and ^13^C NMR data of pinolidoxin (**1**) and bellidisin C.
