# Supplementary material for: Stereoselective Total Synthesis of Natural Decanolides Bellidisin C and Pinolidoxin
Source: Molecules. 2024 Nov 21;29(23):5500. doi: 10.3390/molecules29235500 (PMC11643441; doi:10.3390/molecules29235500)
Supplement: Supplementary file 1 [file molecules-29-05500-s001.zip › molecules-3309229-supplementary.pdf]

Supplementary Materials

File S1

Stereoselective Total Synthesis of Natural Decanolides Bellidisin C and Pinolidoxin

Jingjing Bi [1,5,*], Minhao Chen [2,5], Pengpeng Nie [2,3,4], Yuanfang Liu [2,3,4], Jun Liu [2,3,4,*] and Yuguo Du [2,3,4]

1 School of Pharmacy, Xinyang Agriculture and Forestry University, Xinyang 464000, China; bijingjing2008@126.com
2 State Key Laboratory of Environmental Chemistry and Eco-toxicology, Research Center for Eco-Environmental Sciences, Chinese Academy of Sciences, Beijing 100085, China; a17337704506@163.com (M.C.); niepengpeng20@mails.ucas.ac.cn (P.N.); yuanfangl@126.com (Y.L.); junliu@rcees.ac.cn (J.L.); duyuguo@rcees.ac.cn (Y.D.)
3 State Key Laboratory of Environmental Chemistry and Eco-toxicology, Research Center for Eco-Environmental Sciences, Chinese Academy of Sciences, Beijing 100085, China
4 Binzhou Institute of Technology, Weiqiao-UCAS Science and Technology Park, Binzhou 256606, China
5 School of Chemistry and Chemical Engineering, Henan Normal University, Xinxiang 453007, China
* Correspondence: bijingjing2008@126.com (J.B.); junliu@rcees.ac.cn (J.L.)

Contents

1. Copies of NMR Spectra……………………………………………………..….……………S3

2. Copies of IR Spectra ……………………………………………..………..…..……………S20

3. Comparison of ^{1}H and ^{13}C NMR data of pinolidoxin (**1**) and bellidisin C (**2**) ….…….……S24

1. Copies of NMR Spectra

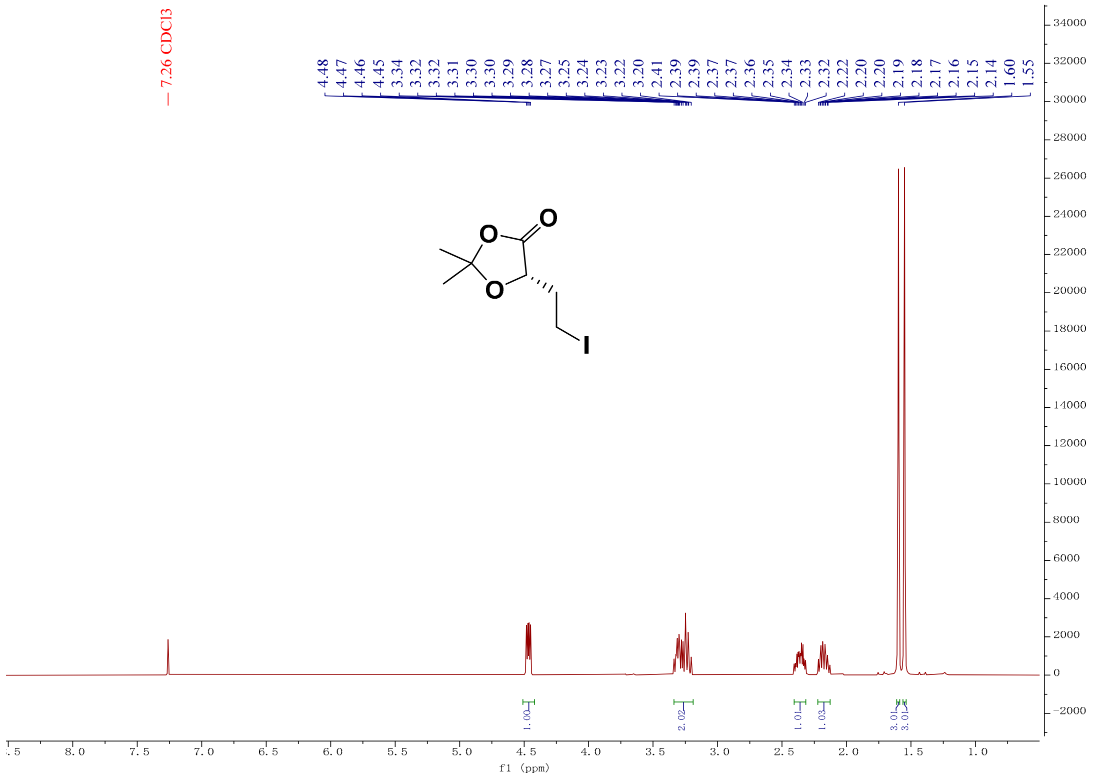

Figure S1. ^{1}H NMR (400 MHz, $CDCl_3$) spectrum of **11**.

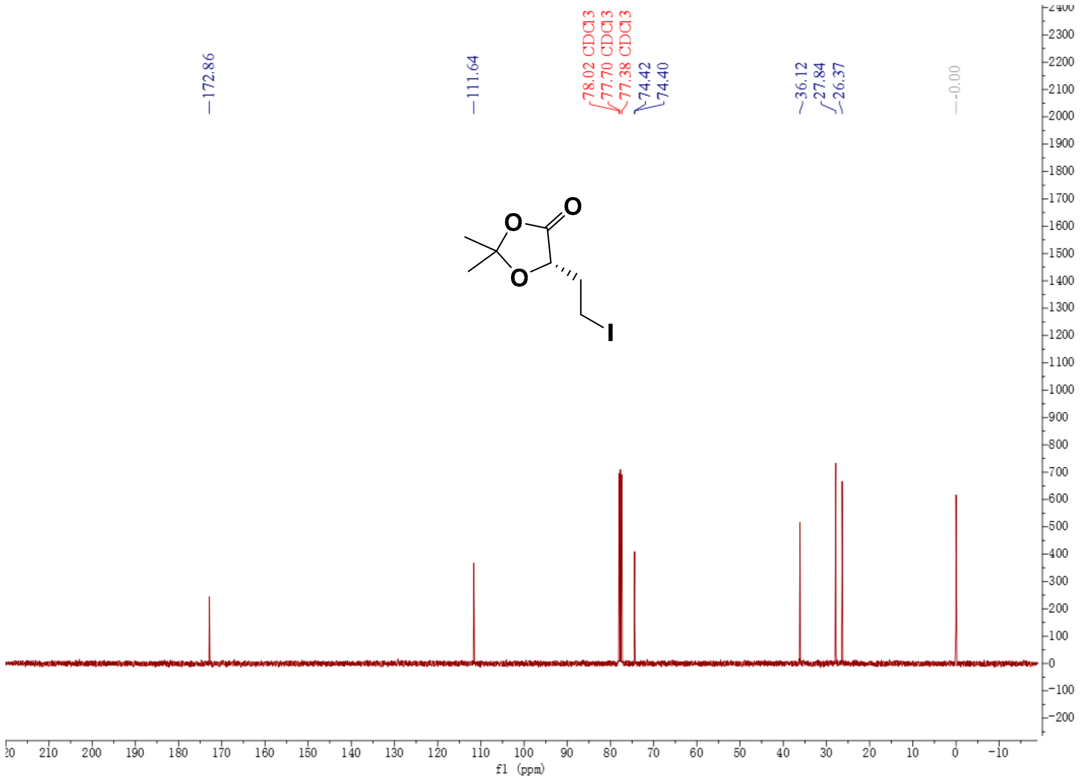

Figure S2. ^{13}C NMR (100 MHz, $CDCl_3$) spectrum of **11**.

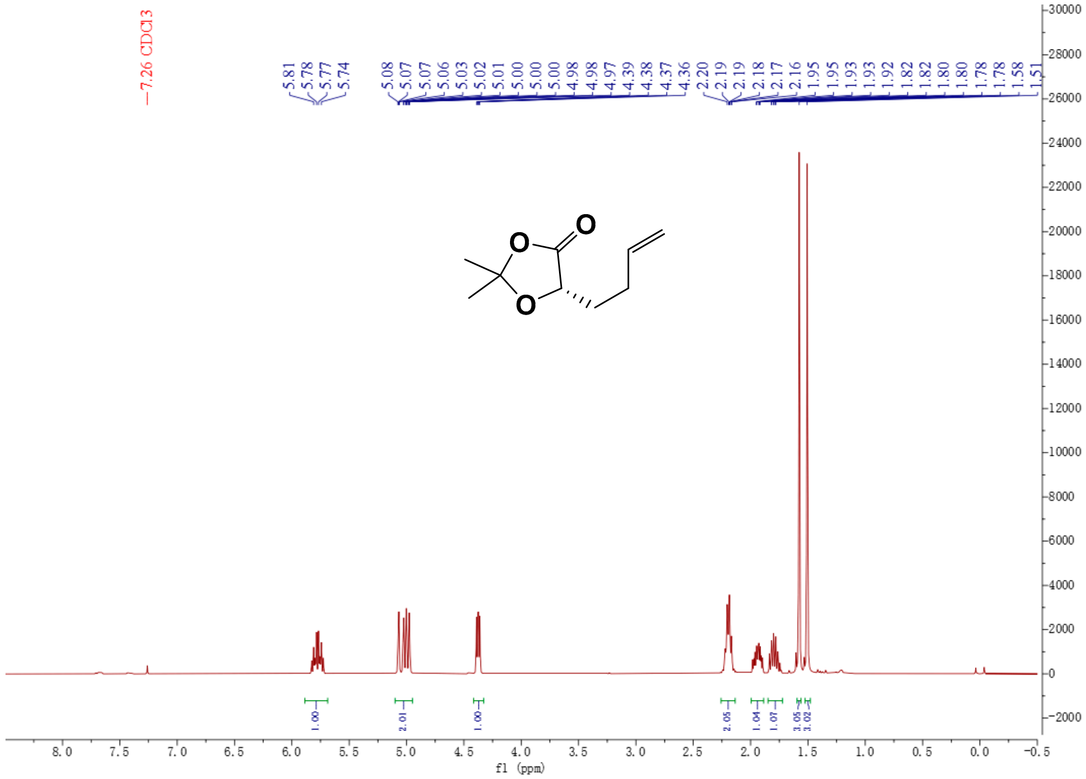

Figure S3. ^{1}H NMR (400 MHz, $CDCl_3$) spectrum of **8**.

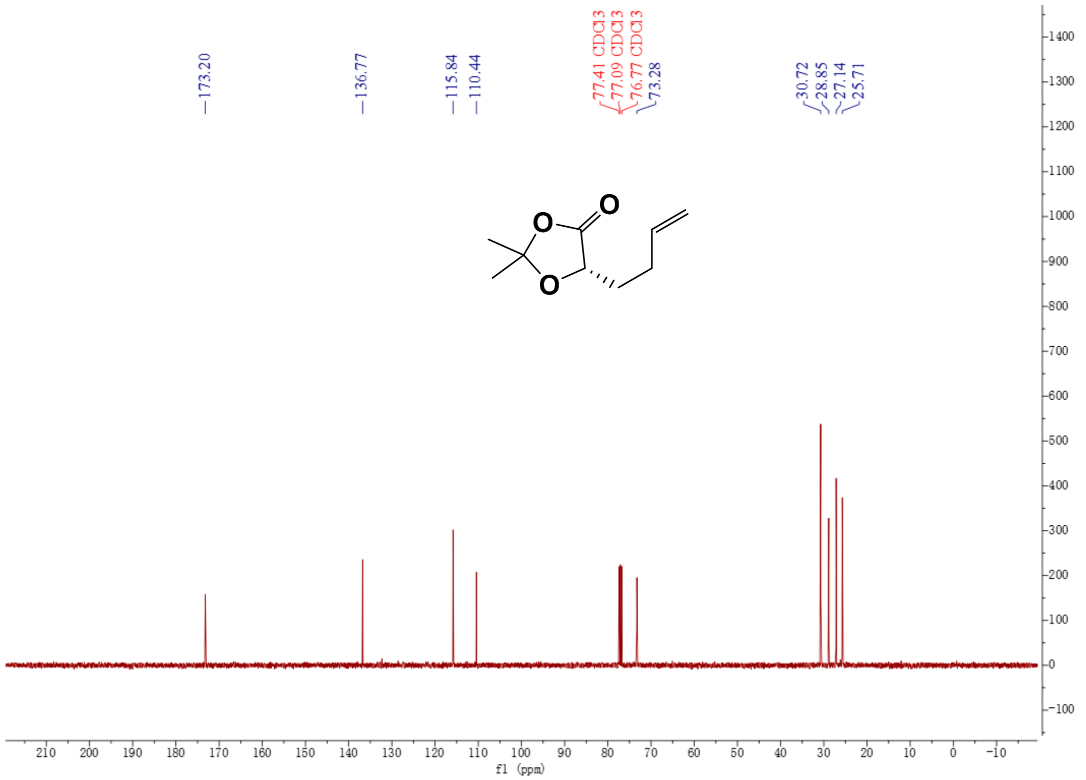

Figure S4. ^{13}C NMR (100 MHz, $CDCl_3$) spectrum of **8**.

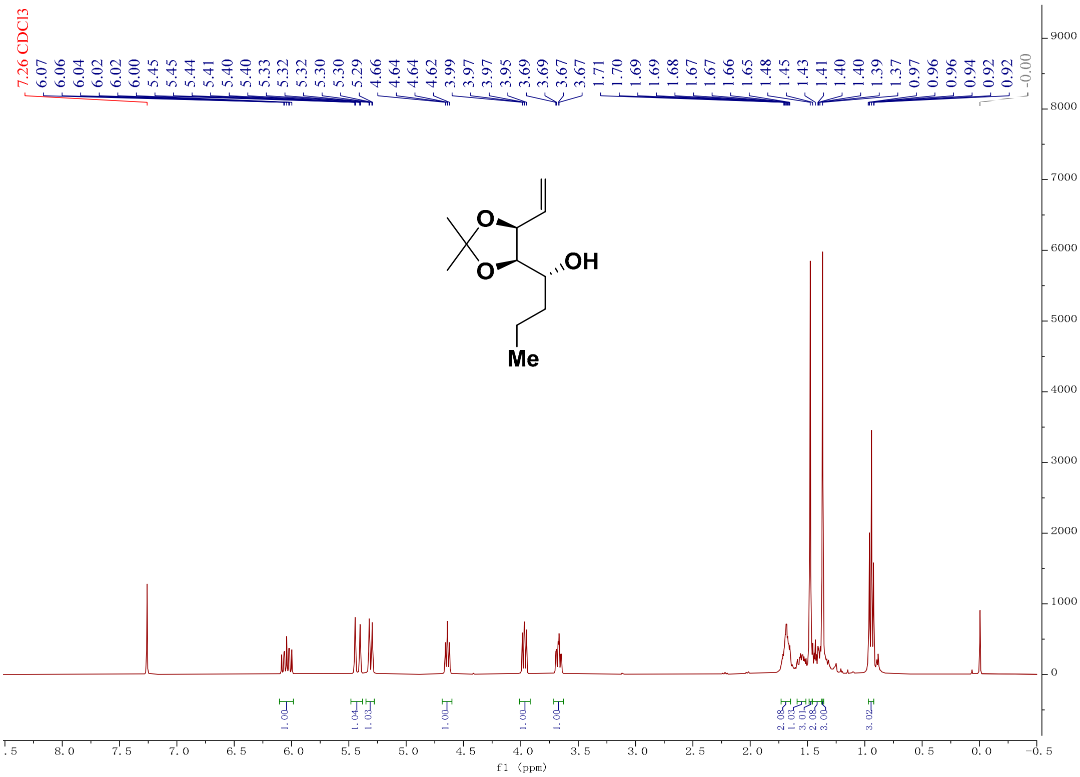

Figure S5. ^{1}H NMR (400 MHz, $CDCl_3$) spectrum of **7a**.

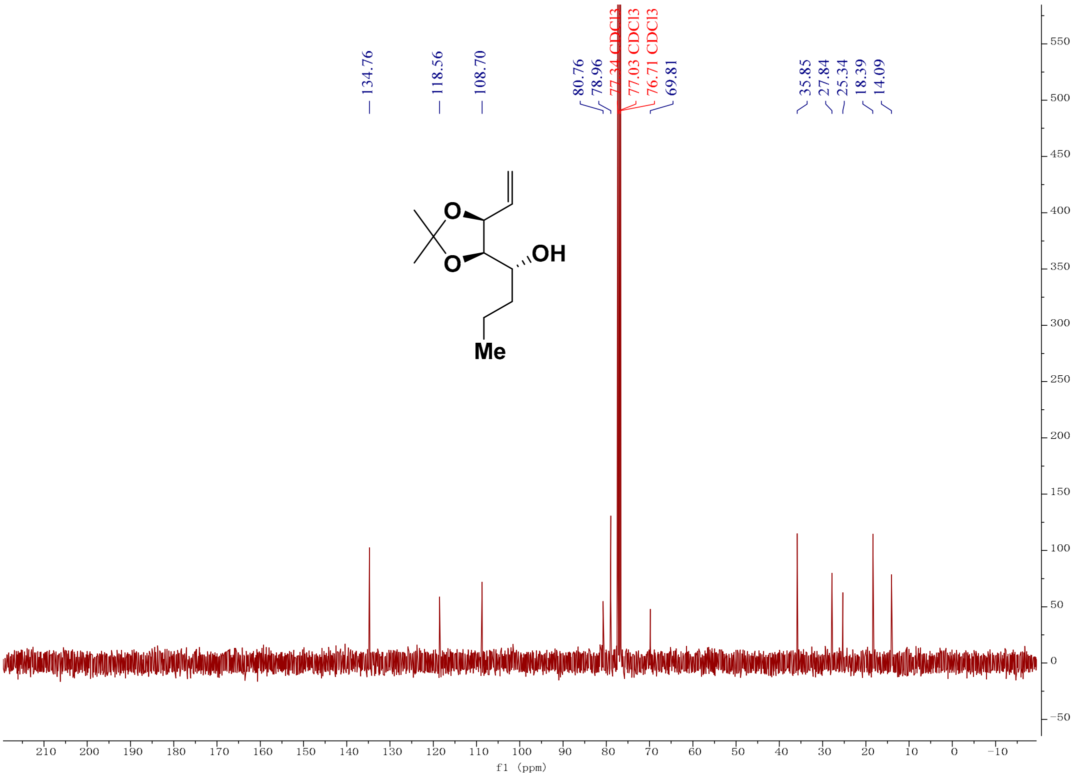

Figure S6. ^{13}C NMR (100 MHz, $CDCl_3$) spectrum of **7a**.

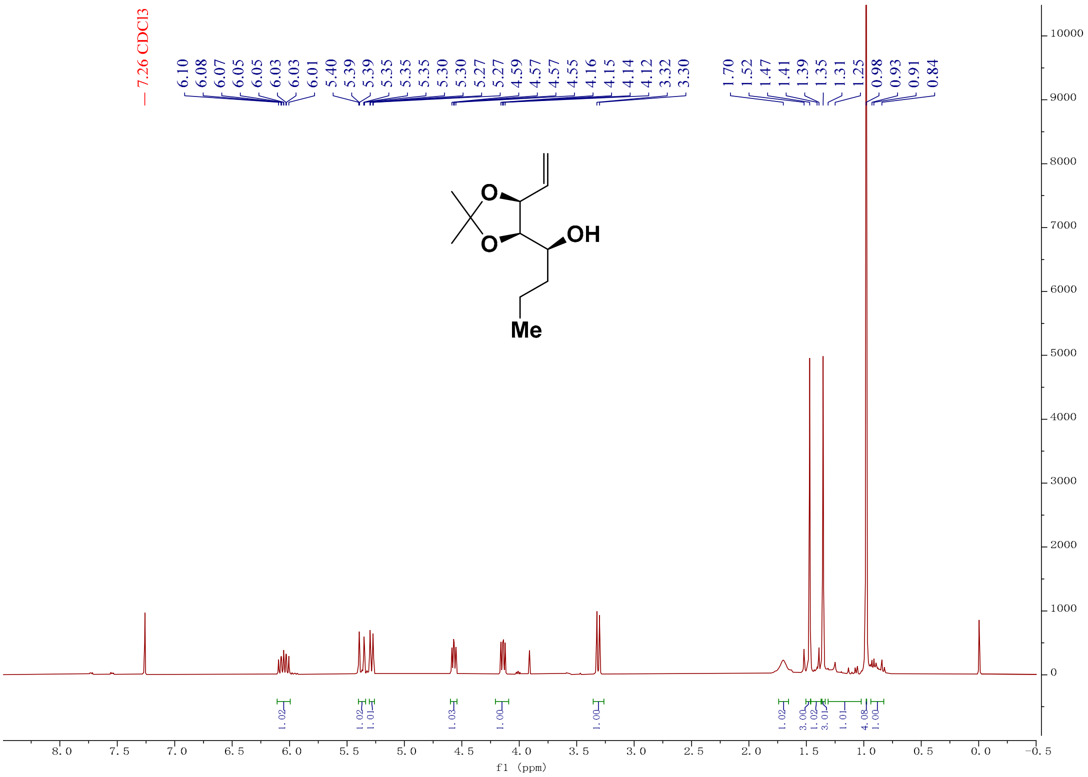

Figure S7. ^{1}H NMR (400 MHz, $CDCl_3$) spectrum of *cis*-**7a**.

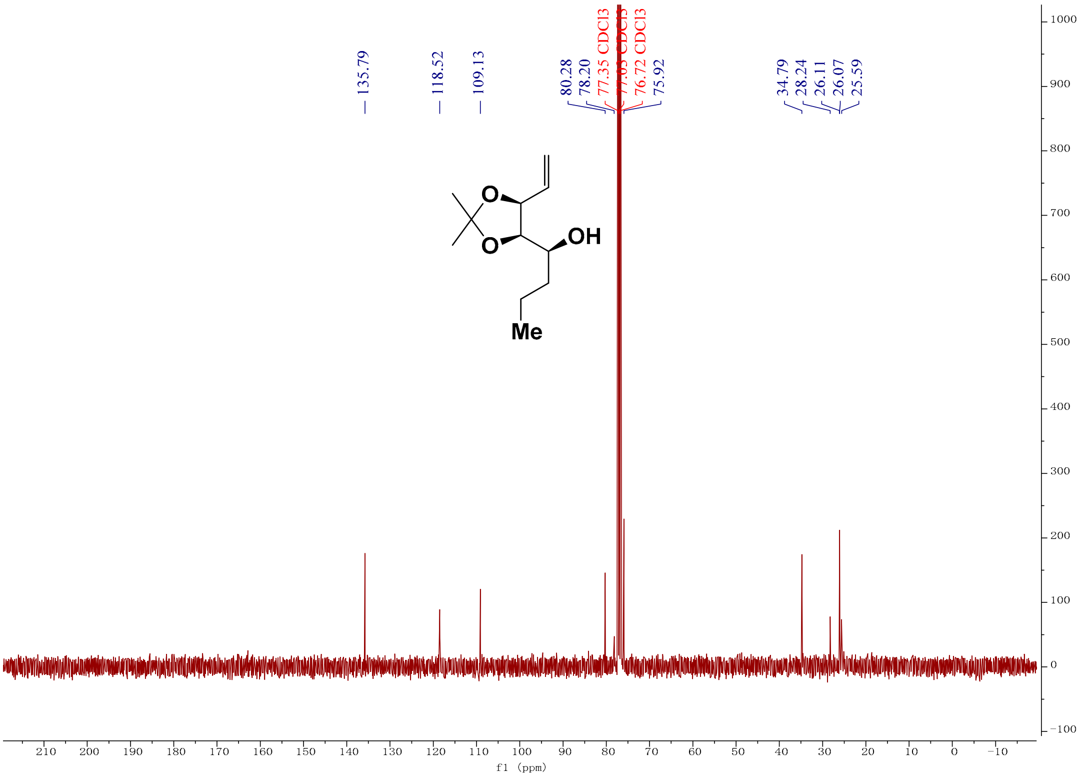

Figure S8. ^{13}C NMR (100 MHz, $CDCl_3$) spectrum of *cis*-**7a**.

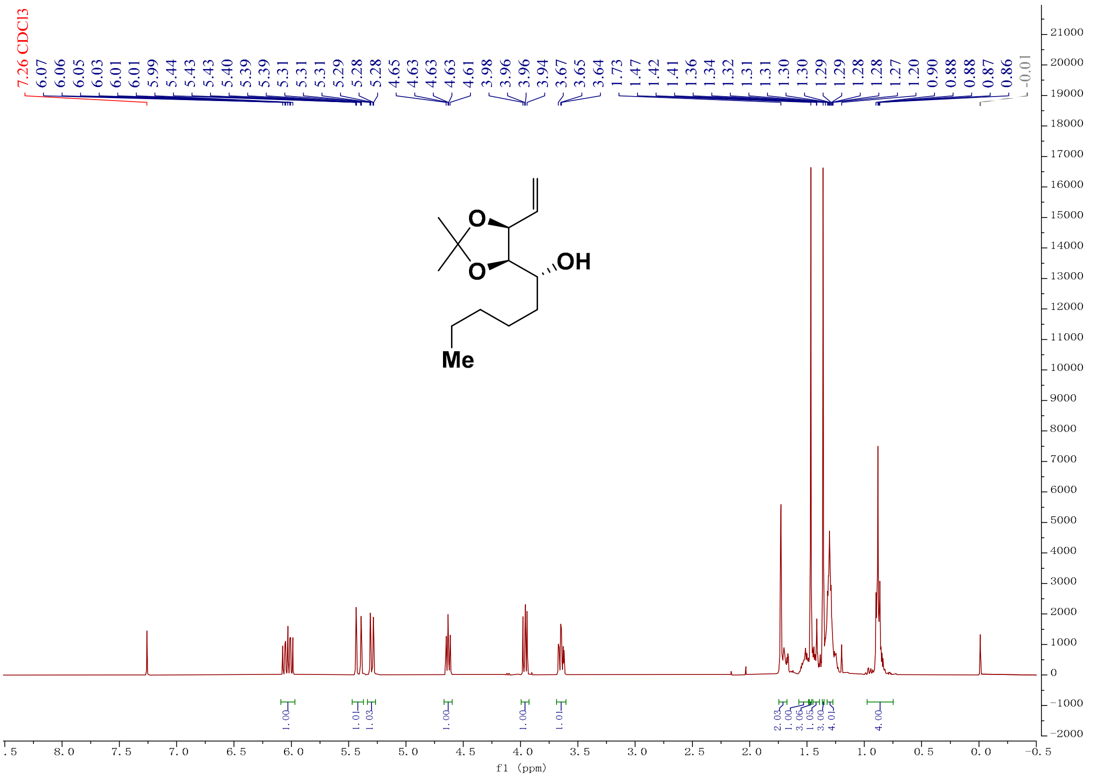

Figure S9. ^{1}H NMR (400 MHz, $CDCl_3$) spectrum of **7b**.

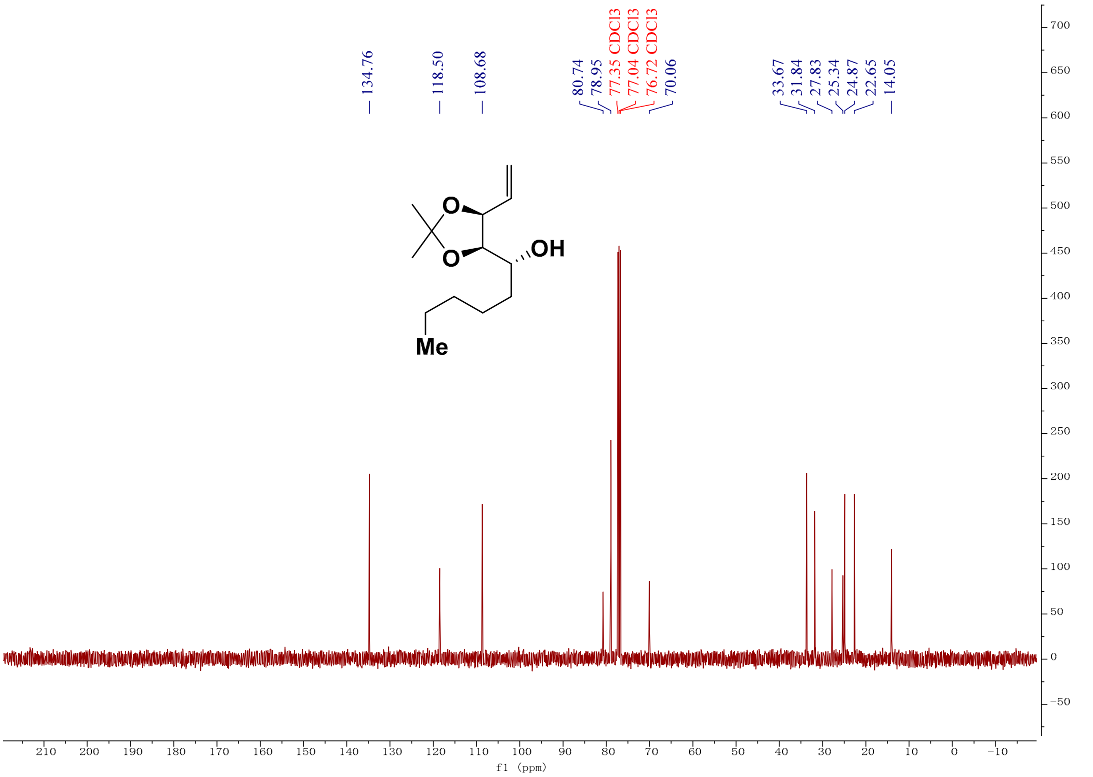

Figure S10. ^{13}C NMR (100 MHz, $CDCl_3$) spectrum of **7b**.

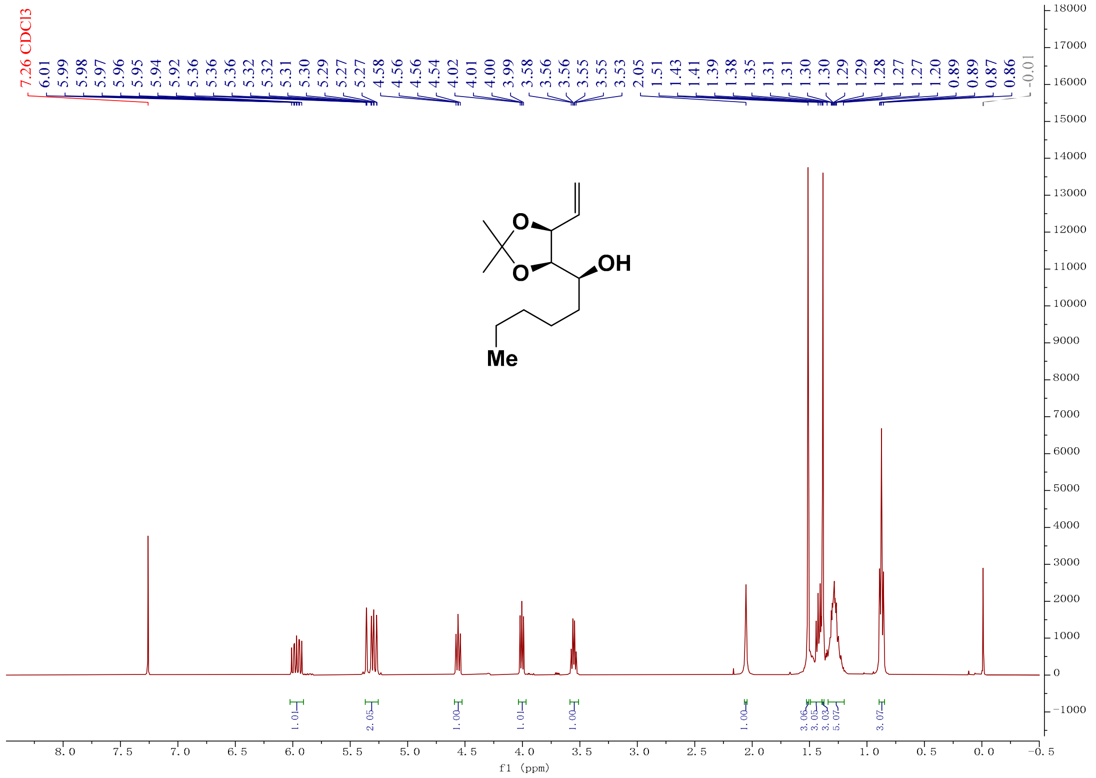

Figure S11. ^{1}H NMR (400 MHz, $CDCl_3$) spectrum of *cis*-**7b**.

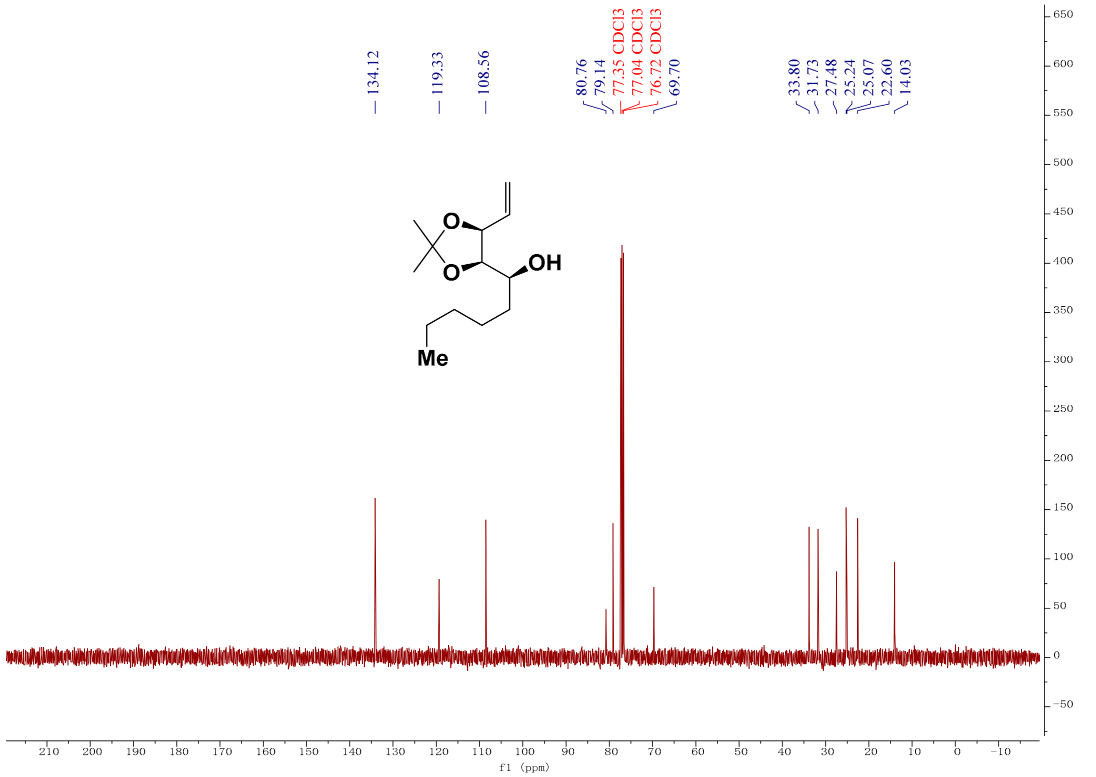

Figure S12. ^{13}C NMR (100 MHz, $CDCl_3$) spectrum of *cis*-**7b**.

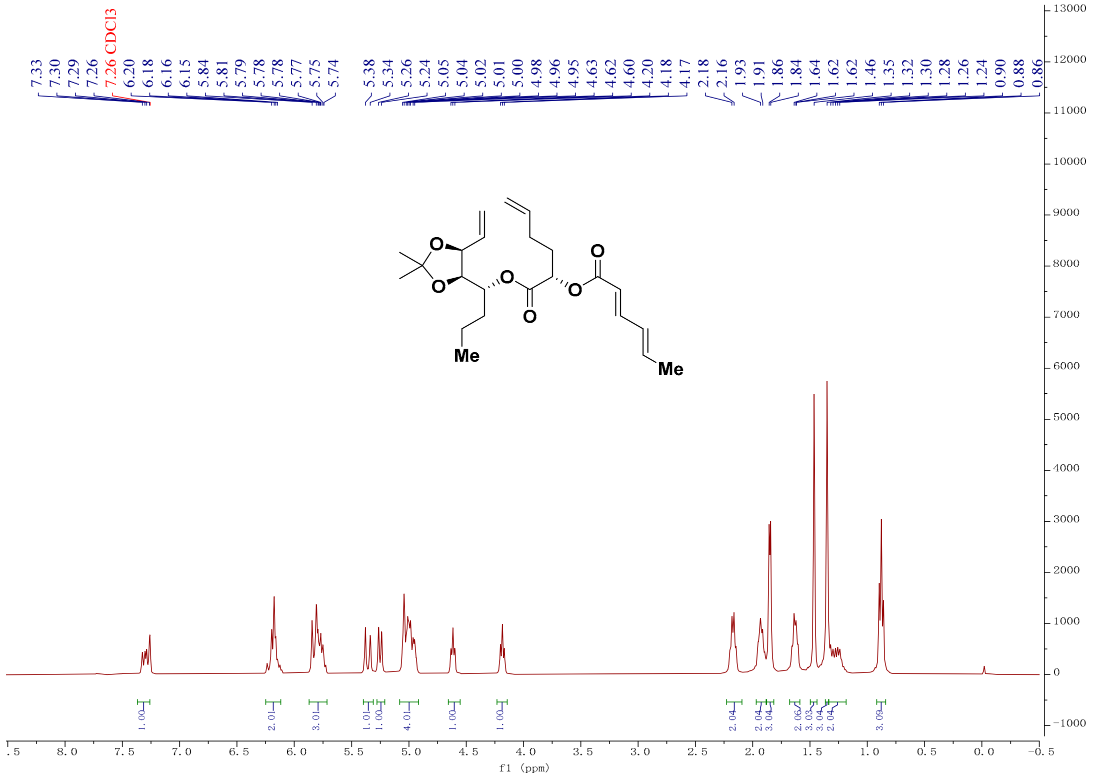

Figure S13. ^{1}H NMR (400 MHz, $CDCl_3$) spectrum of **13a**.

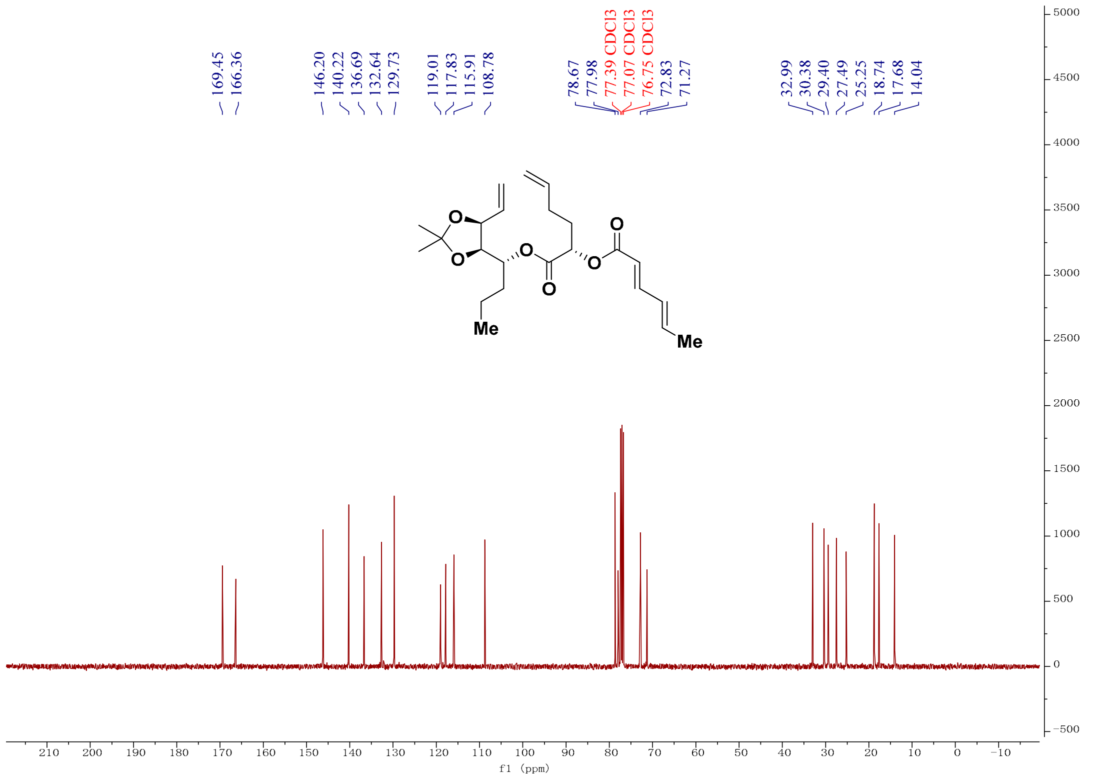

Figure S14. ^{13}C NMR (100 MHz, $CDCl_3$) spectrum of **13a**.

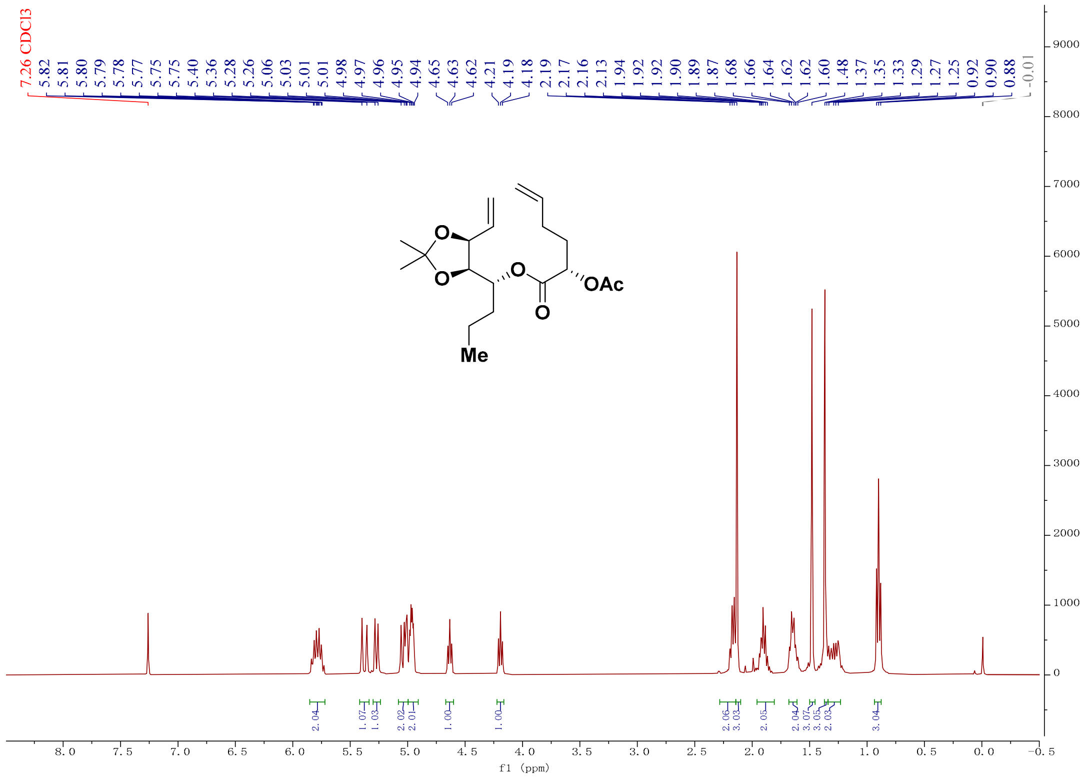

Figure S15. ^{1}H NMR (400 MHz, $CDCl_3$) spectrum of **13b**.

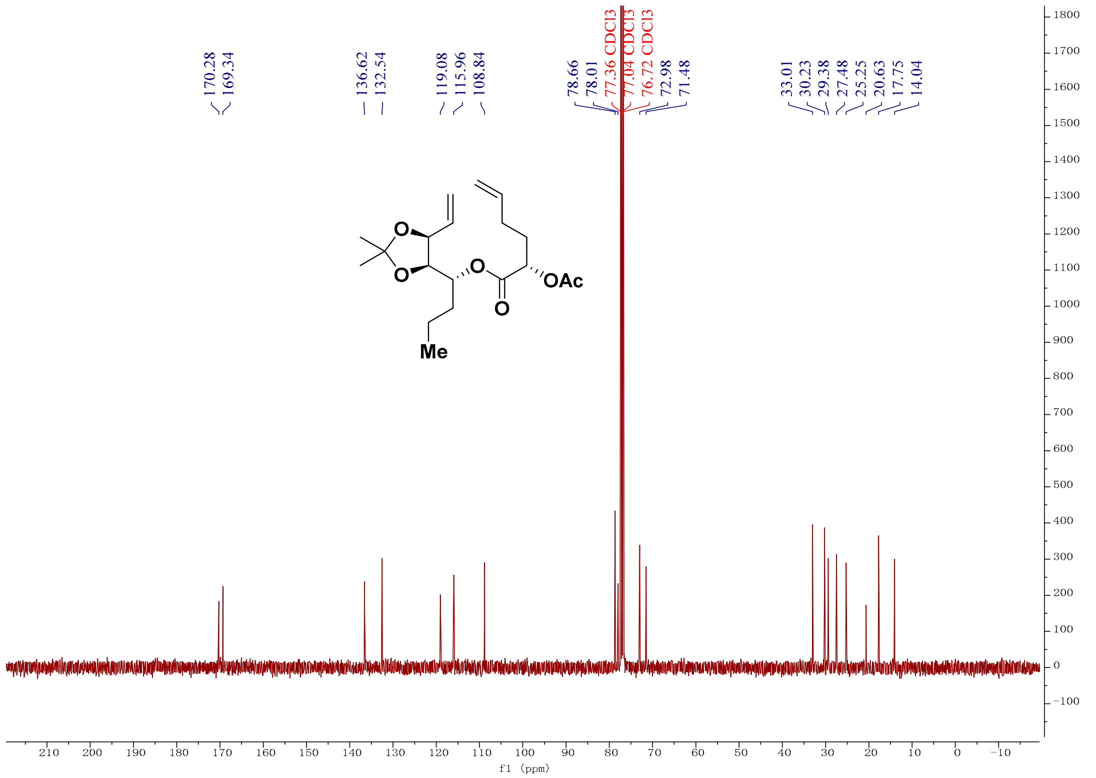

Figure S16. ^{13}C NMR (100 MHz, $CDCl_3$) spectrum of **13b**.

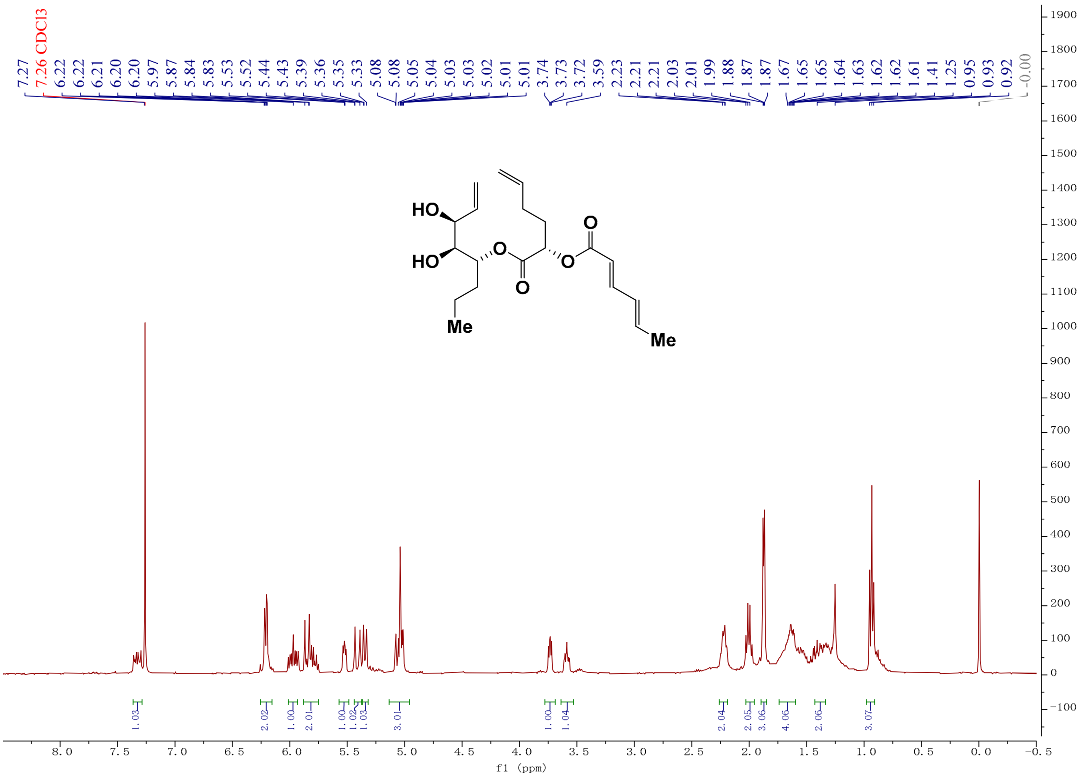

Figure S17. ^{1}H NMR (400 MHz, $CDCl_3$) spectrum of **14**.

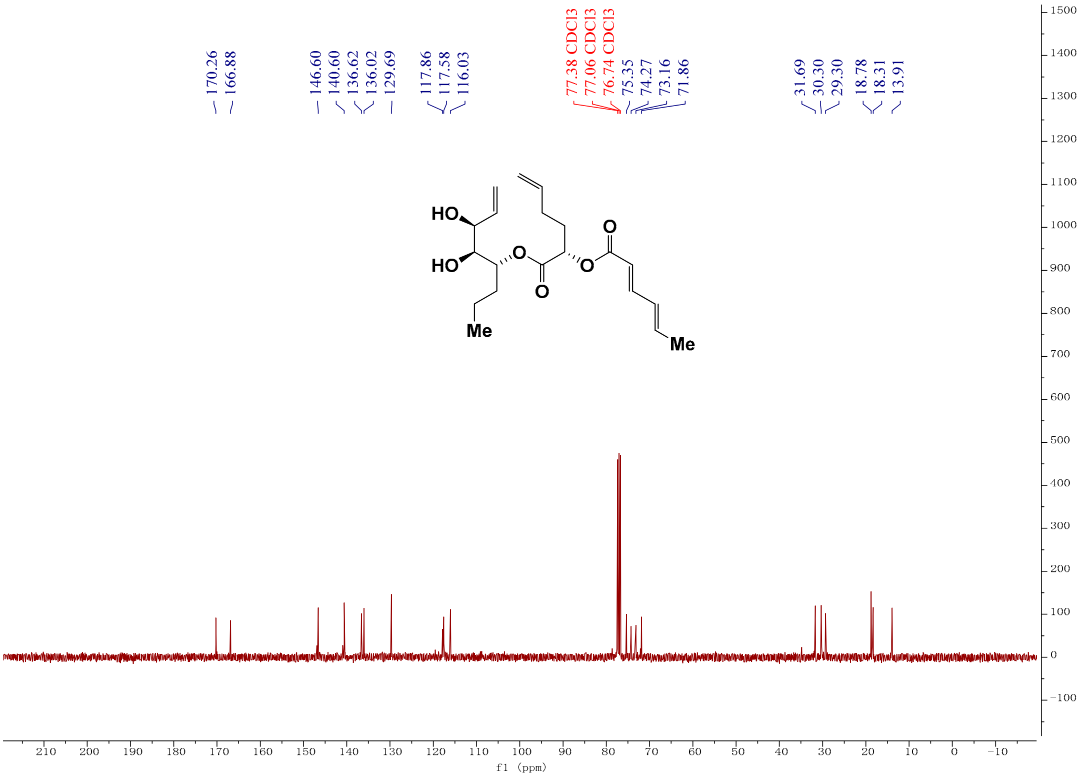

Figure S18. ^{13}C NMR (100 MHz, $CDCl_3$) spectrum of **14**.

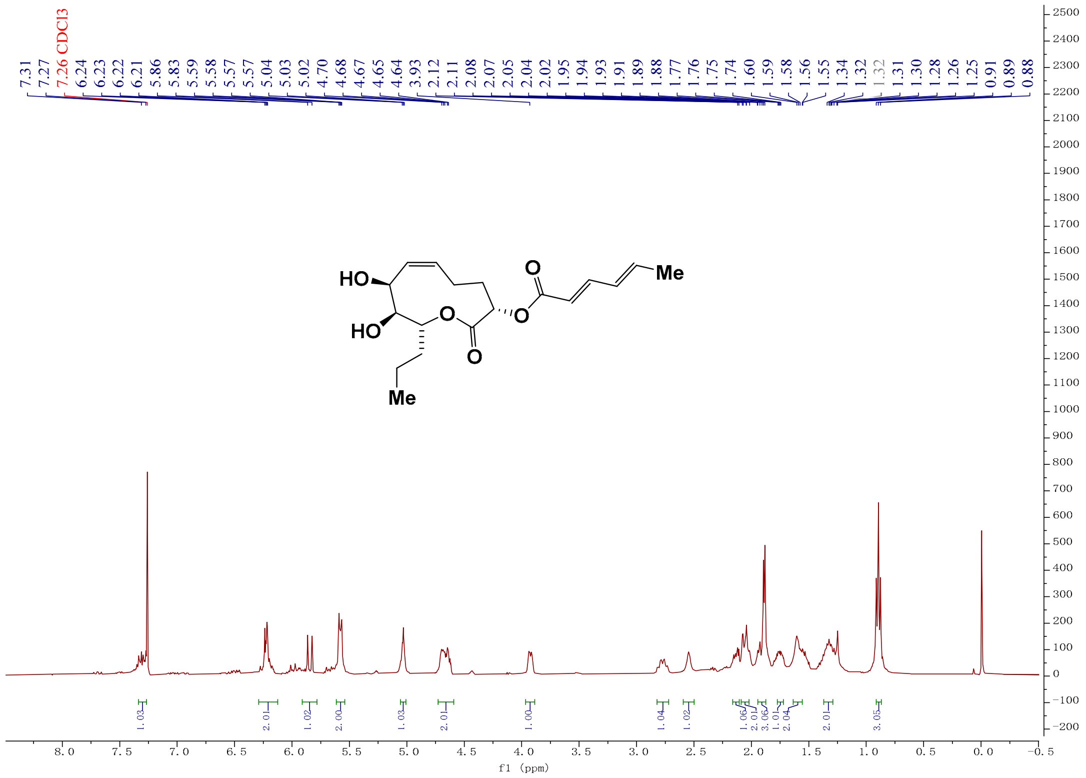

Figure S19. ^{1}H NMR (400 MHz, $CDCl_3$) spectrum of **14a** (Z isomer of pinolidoxin).

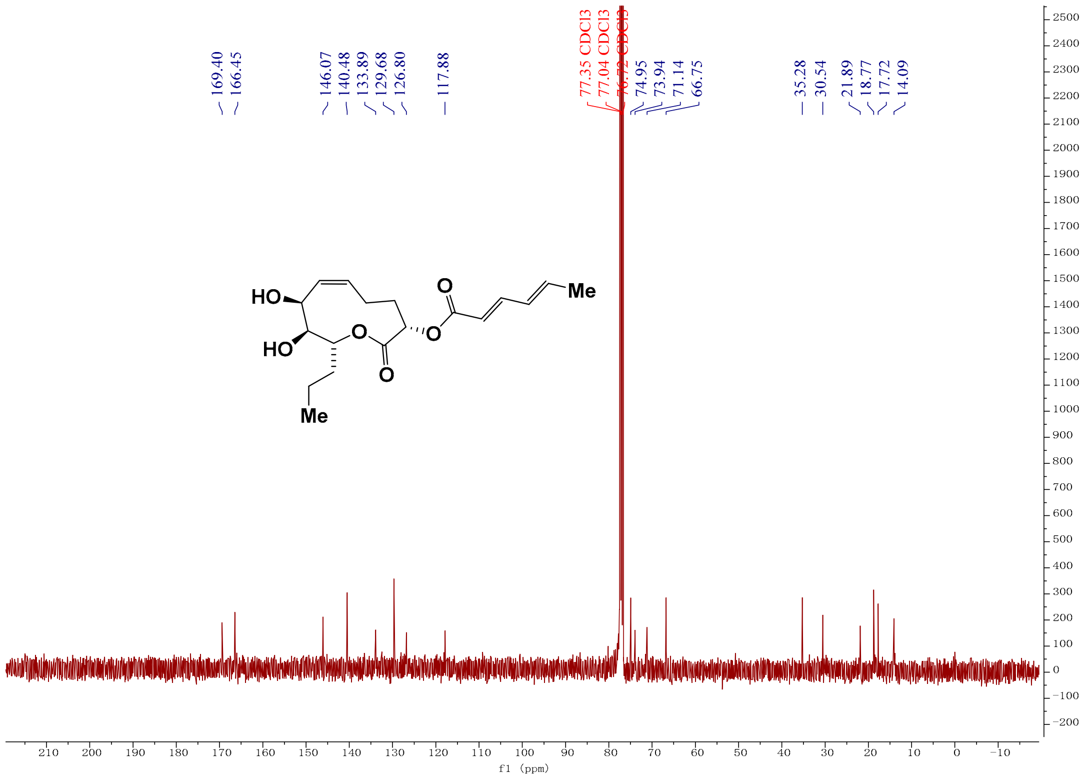

Figure S20. ^{13}C NMR (100 MHz, $CDCl_3$) spectrum of **14a** (Z isomer of pinolidoxin).

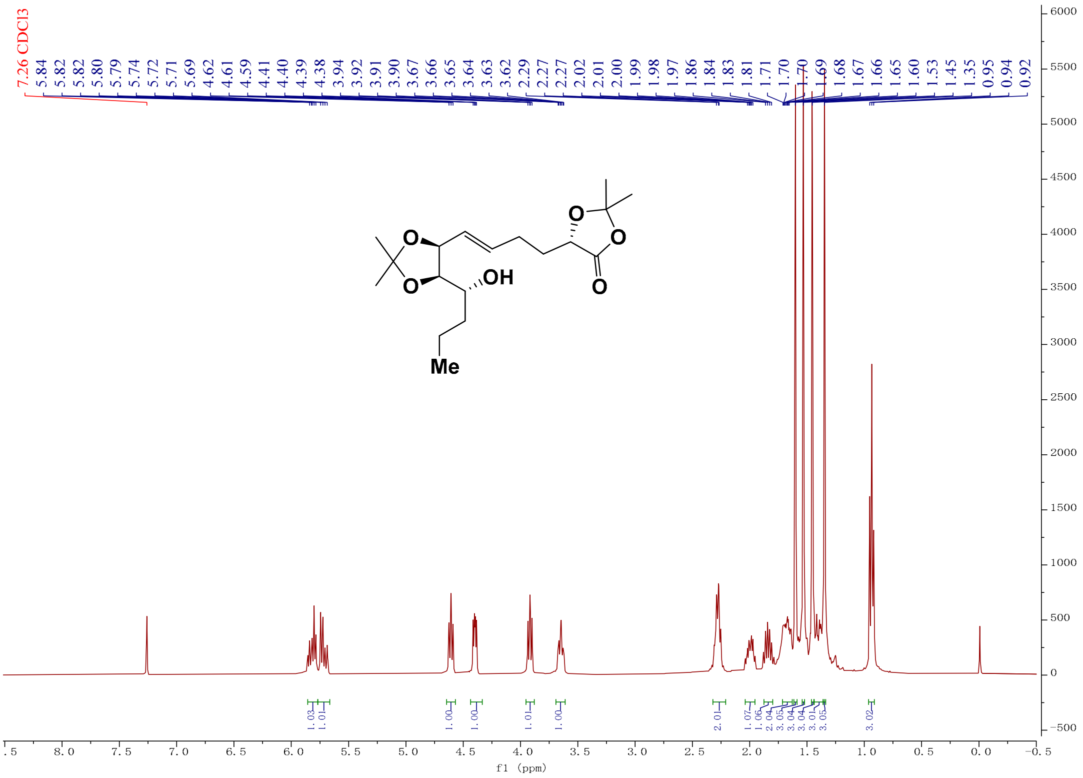

Figure S21. ^{1}H NMR (400 MHz, $CDCl_3$) spectrum of **6a**.

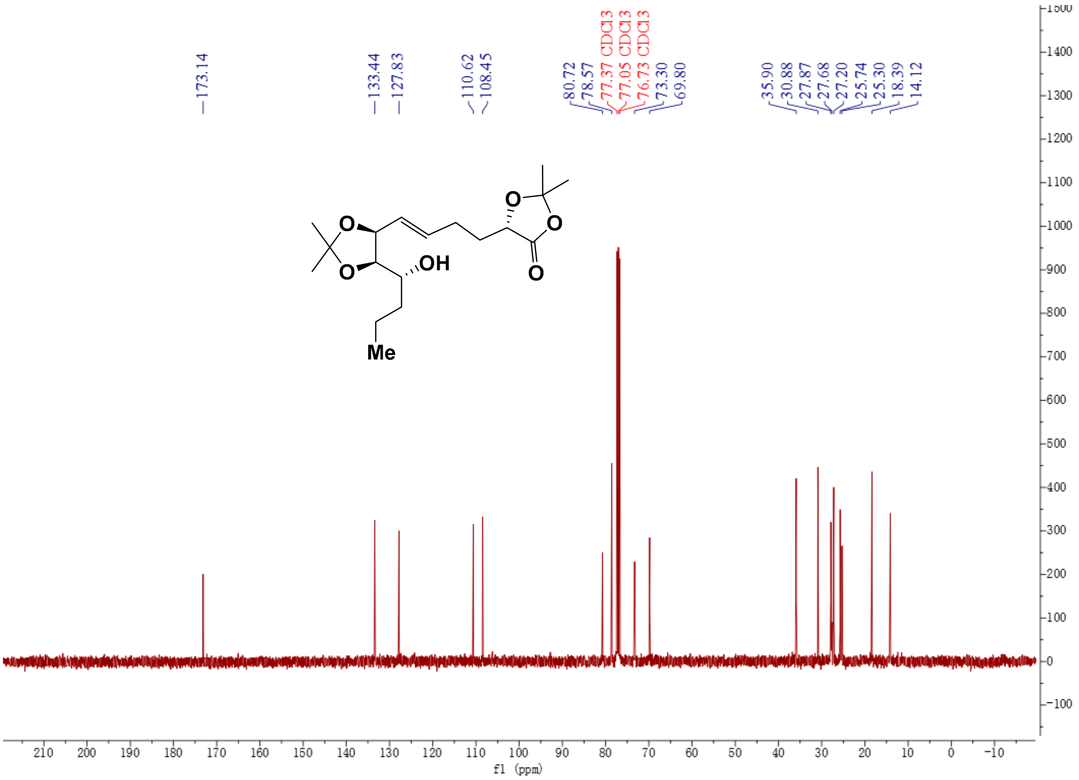

Figure S22. ^{13}C NMR (100 MHz, $CDCl_3$) spectrum of **6a**.

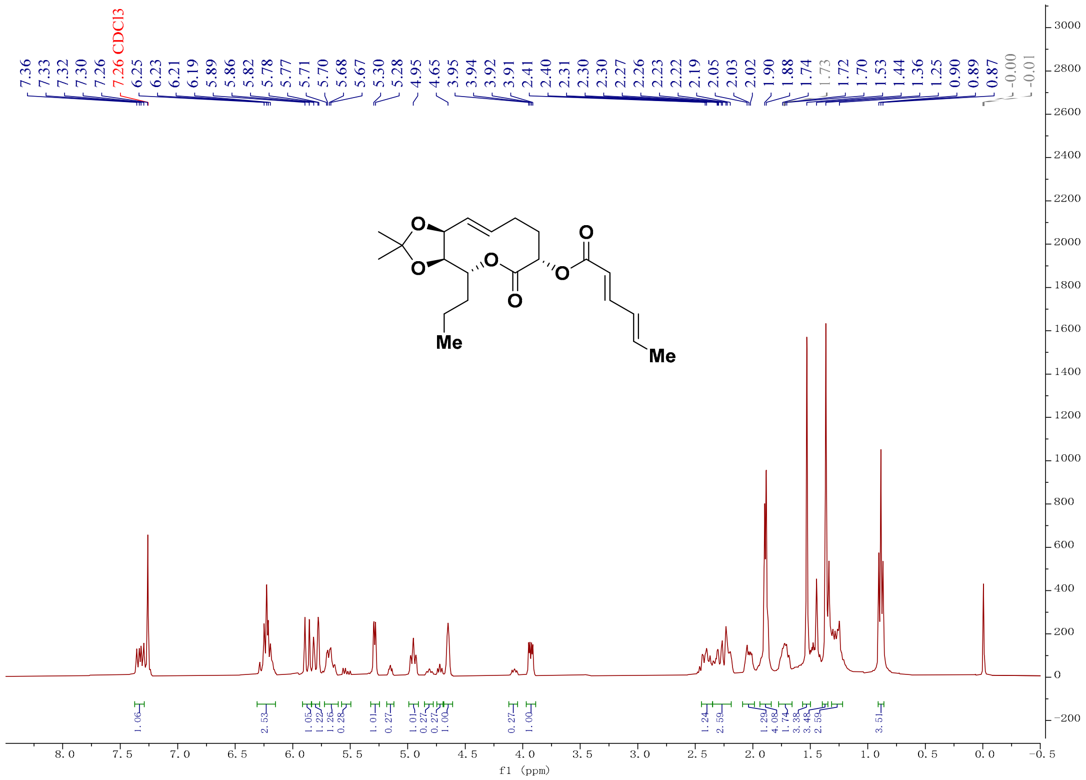

Figure S23. ^{1}H NMR (400 MHz, $CDCl_3$) spectrum of **15** (*with conformational equilibrium*).

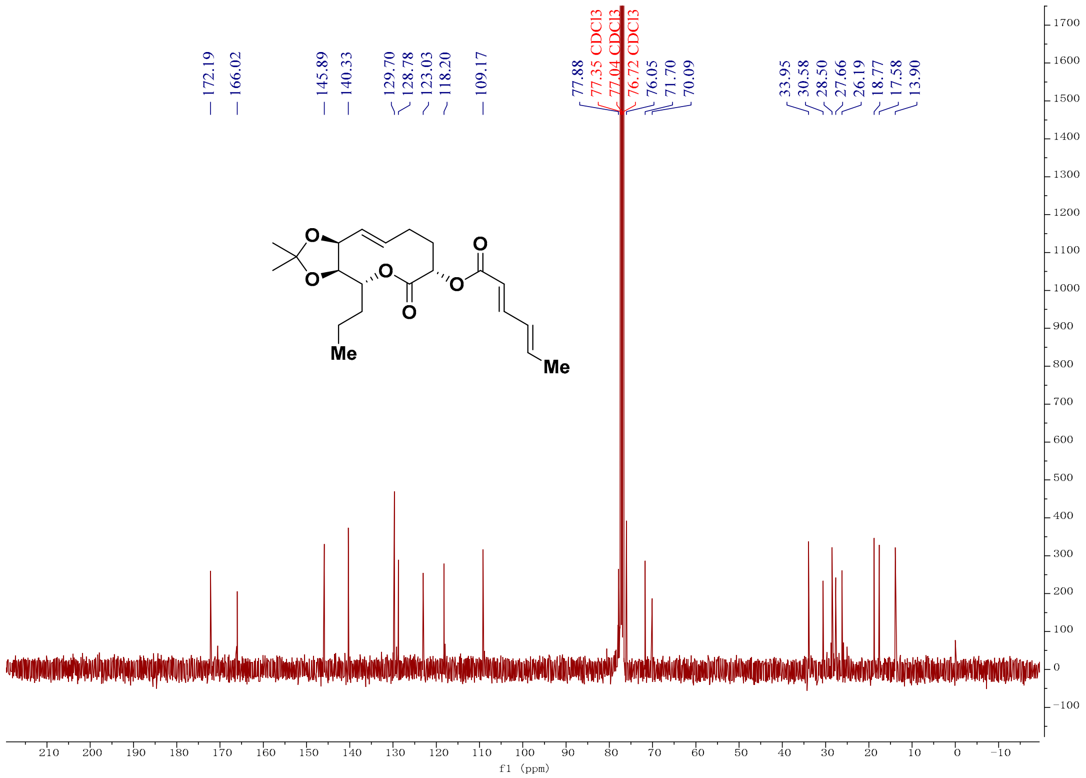

Figure S24. ^{13}C NMR (100 MHz, $CDCl_3$) spectrum of **15** (*with conformational equilibrium*).

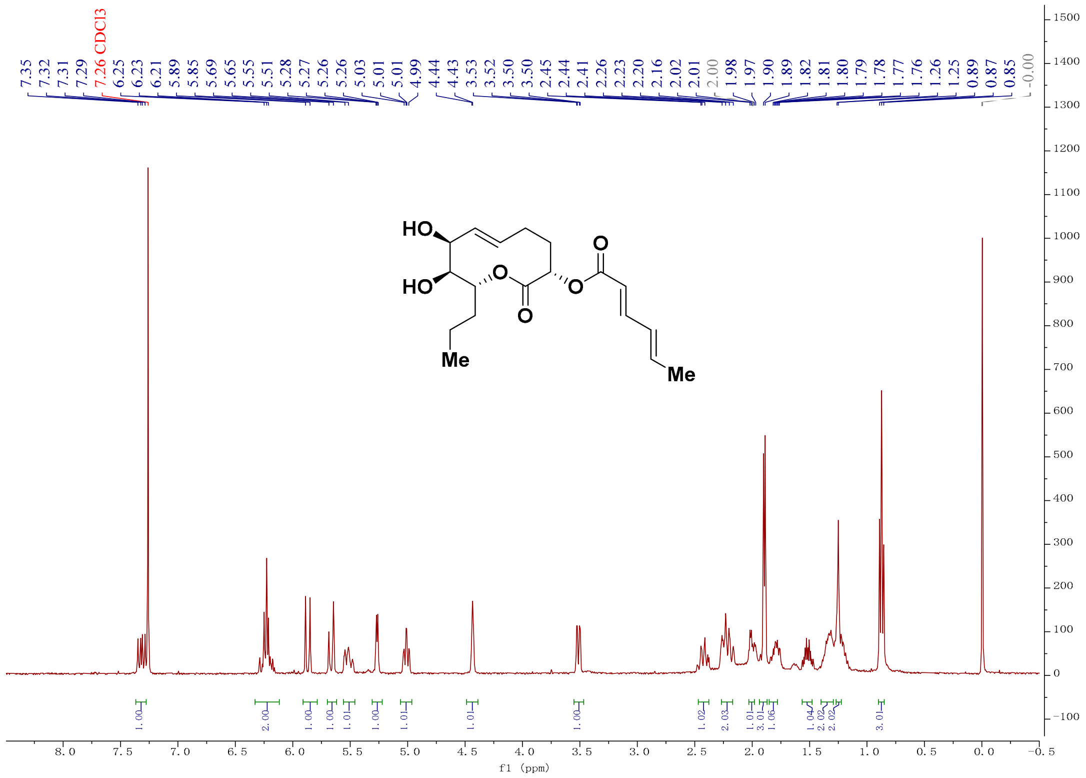

Figure s25. ^{1}H NMR (400 MHz, $CDCl_3$) spectrum of Pinolidoxin (**1**).

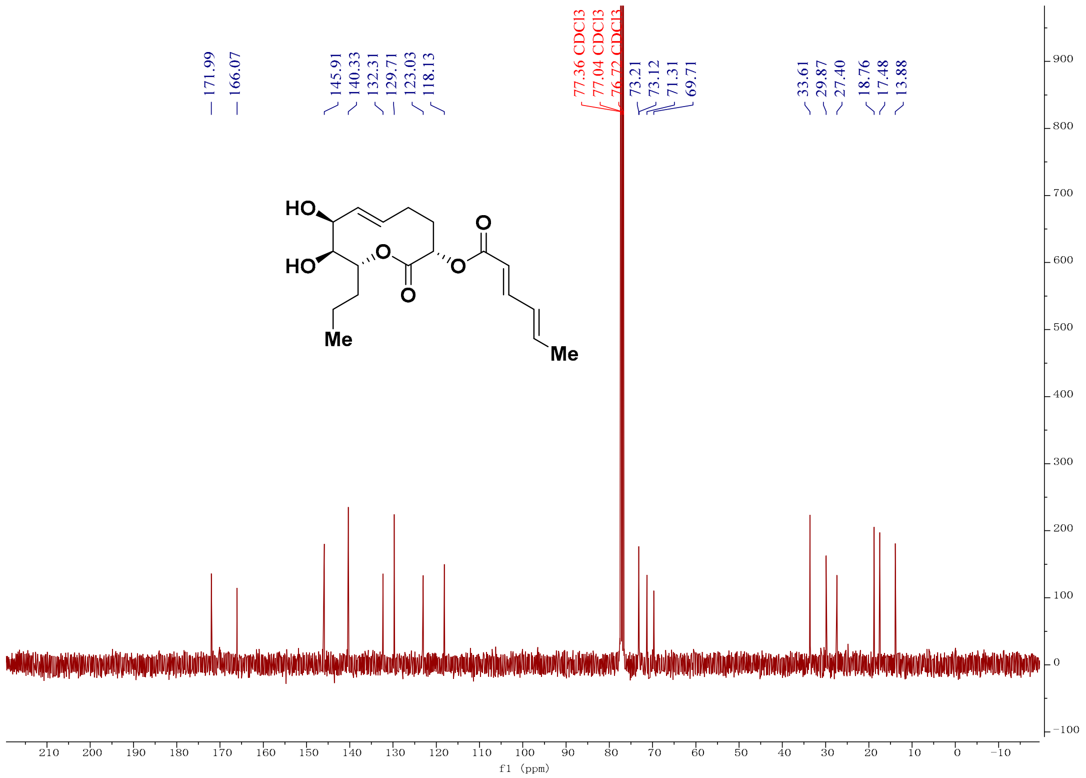

Figure S26. ^{13}C NMR (100 MHz, $CDCl_3$) spectrum of Pinolidoxin (**1**).

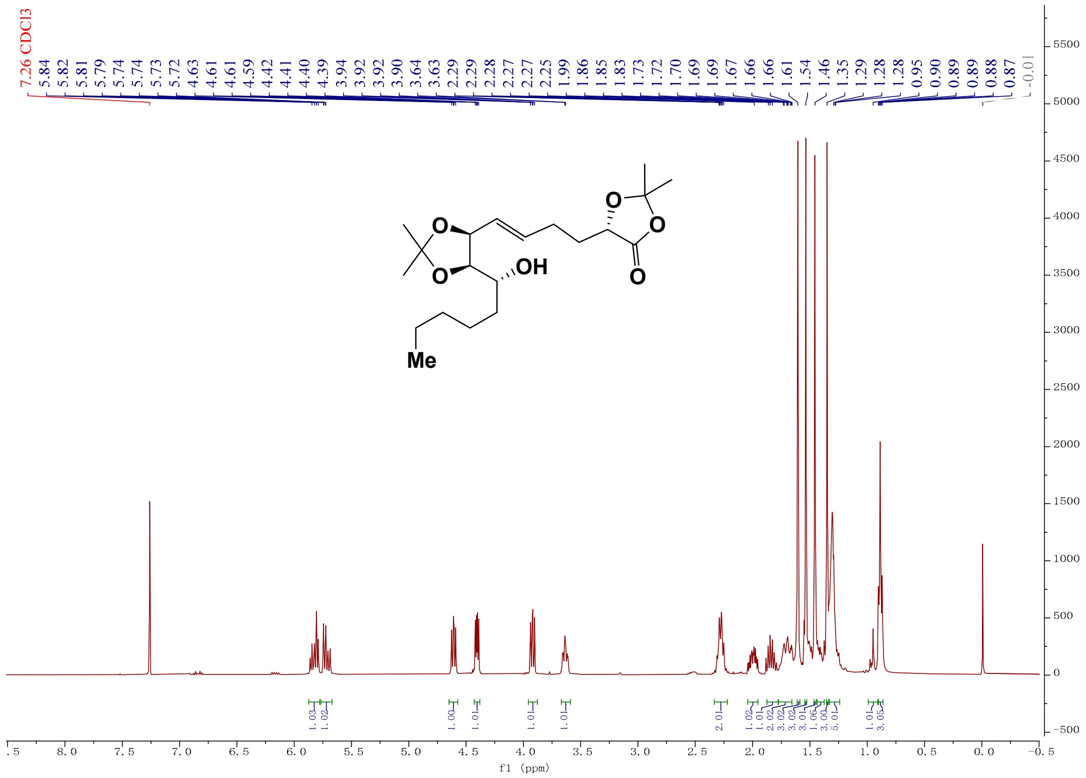

Figure S27. ^{1}H NMR (400 MHz, $CDCl_3$) spectrum of **6b**.

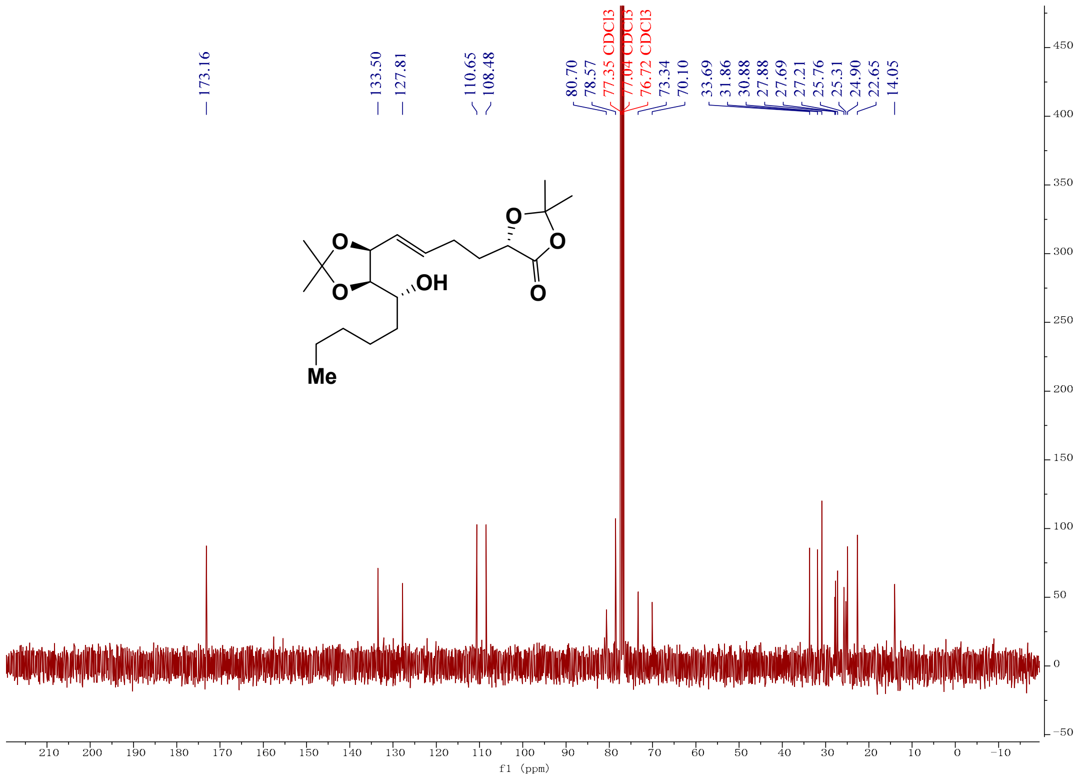

Figure S28. ^{13}C NMR (100 MHz, $CDCl_3$) spectrum of **6b**.

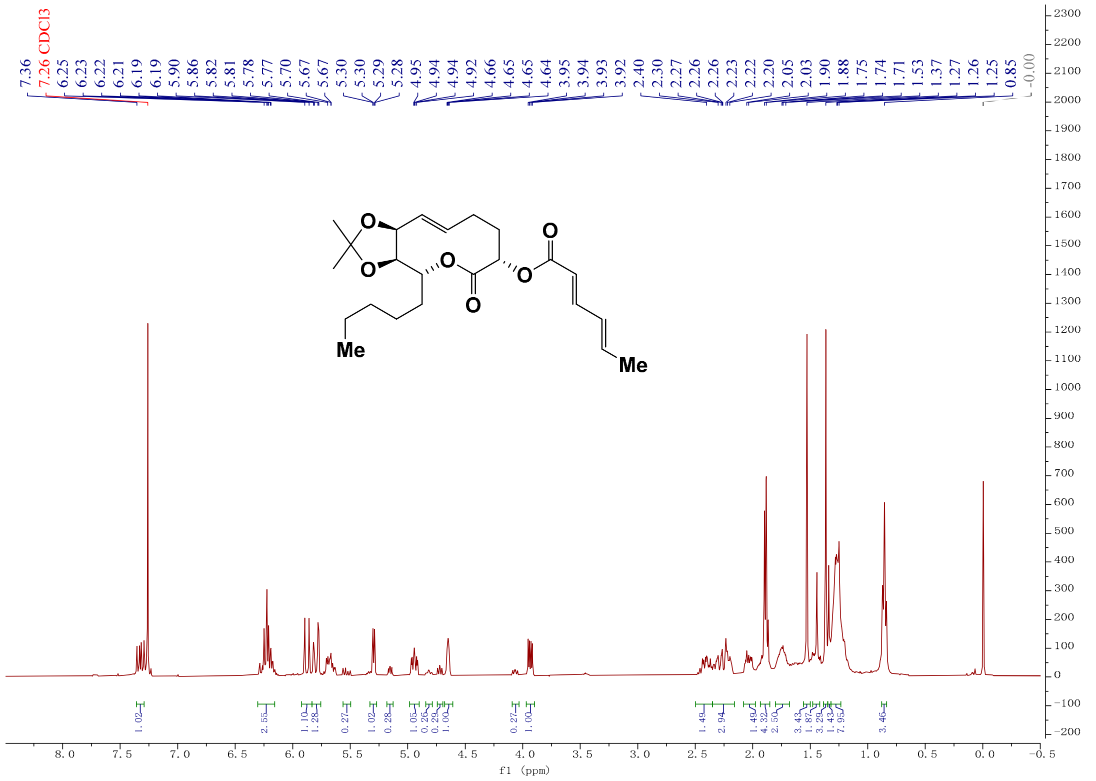

Figure S29. ^{1}H NMR (400 MHz, $CDCl_3$) spectrum of **16** (*with conformational equilibrium*).

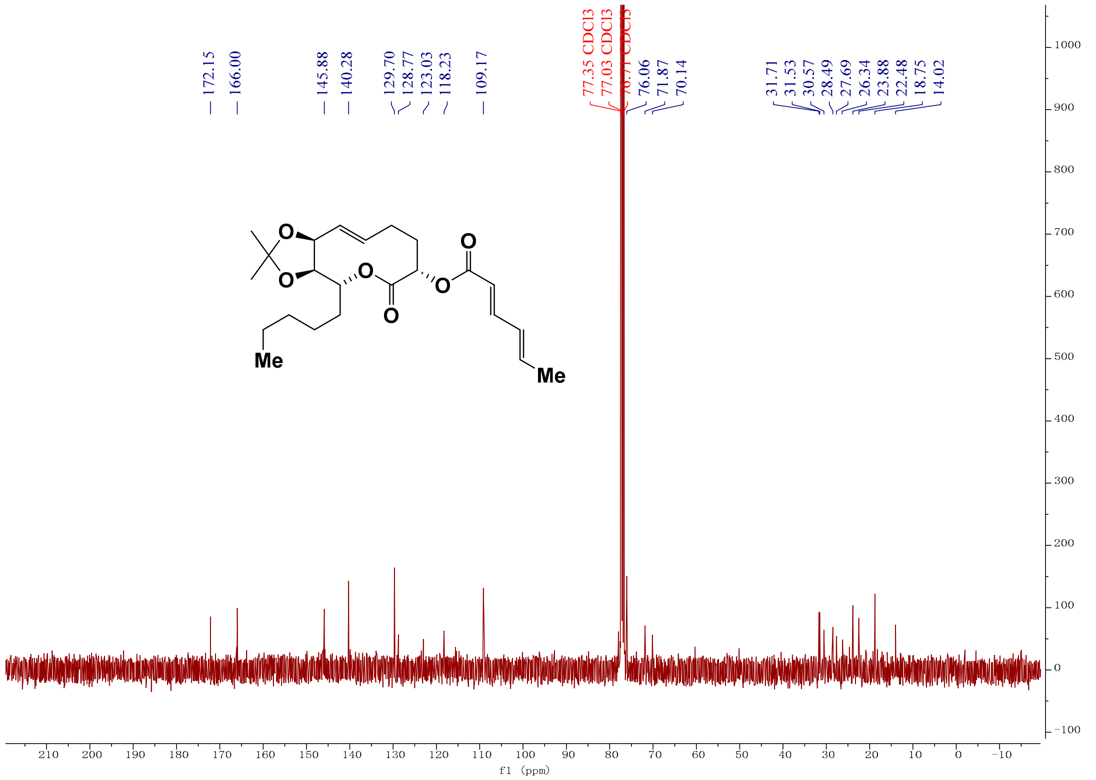

Figure S30. ^{13}C NMR (100 MHz, $CDCl_3$) spectrum of **16** (*with conformational equilibrium*).

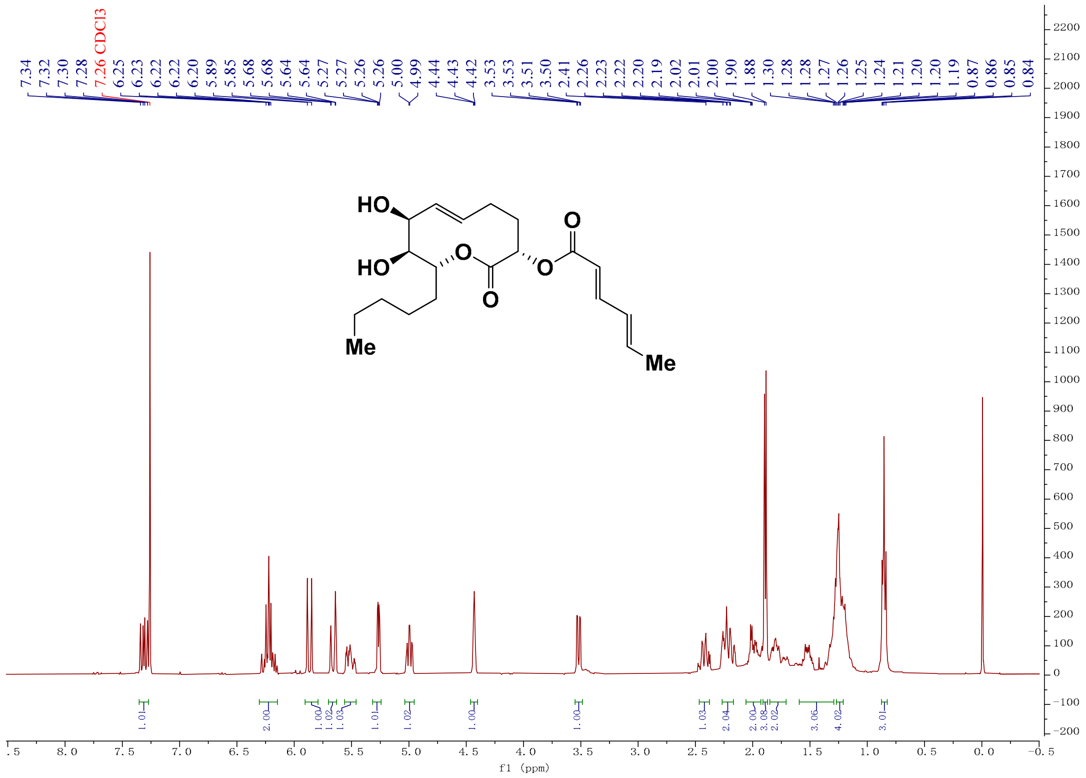

Figure S31. ^{1}H NMR (400 MHz, $CDCl_3$) spectrum of bellidisin C (**2**).

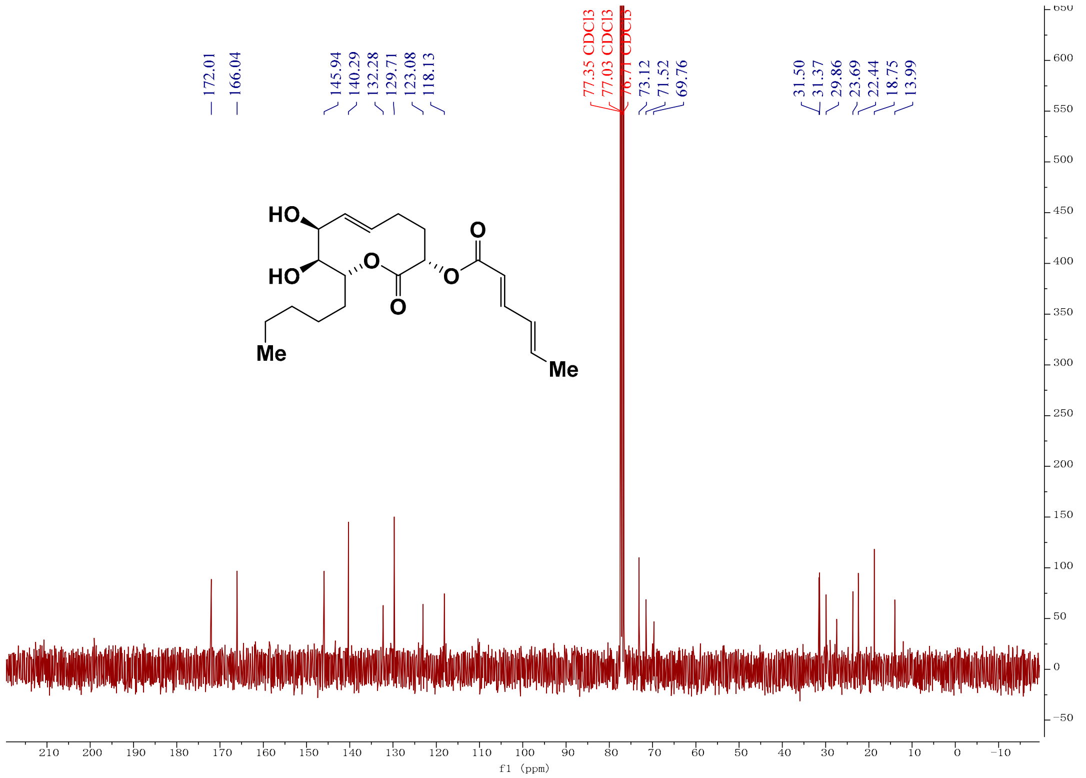

Figure S32. ^{13}C NMR (100 MHz, $CDCl_3$) spectrum of bellidisin C (**2**).

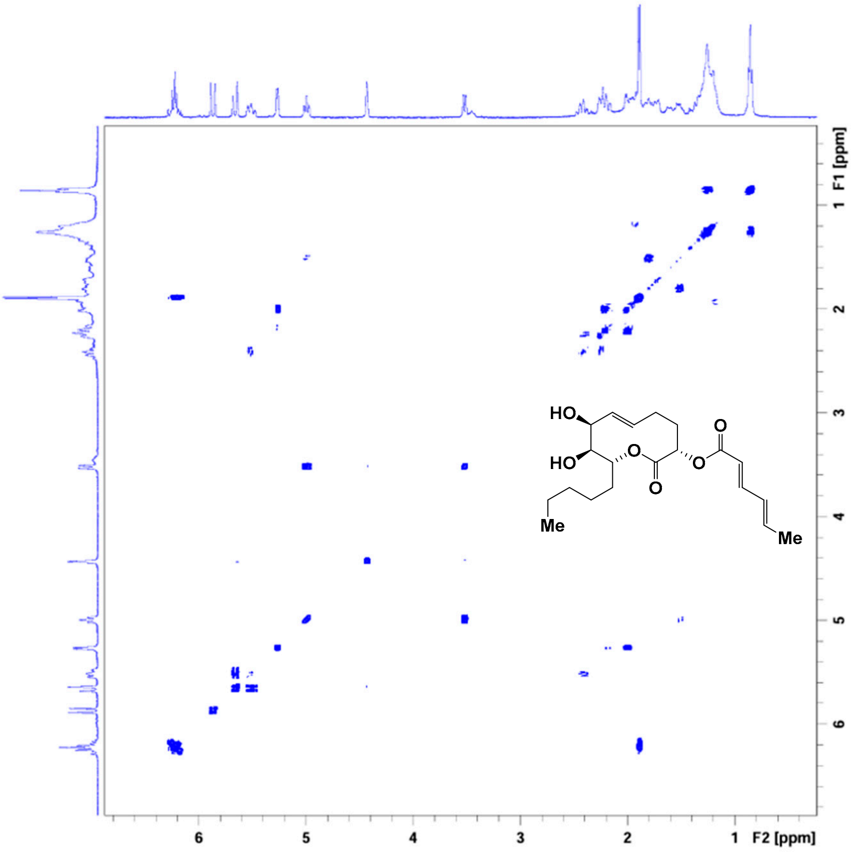

Figure S33. ^{1}H-^{1}H COSY (400 MHz, $CDCl_3$) spectrum of bellidisin C (**2**).

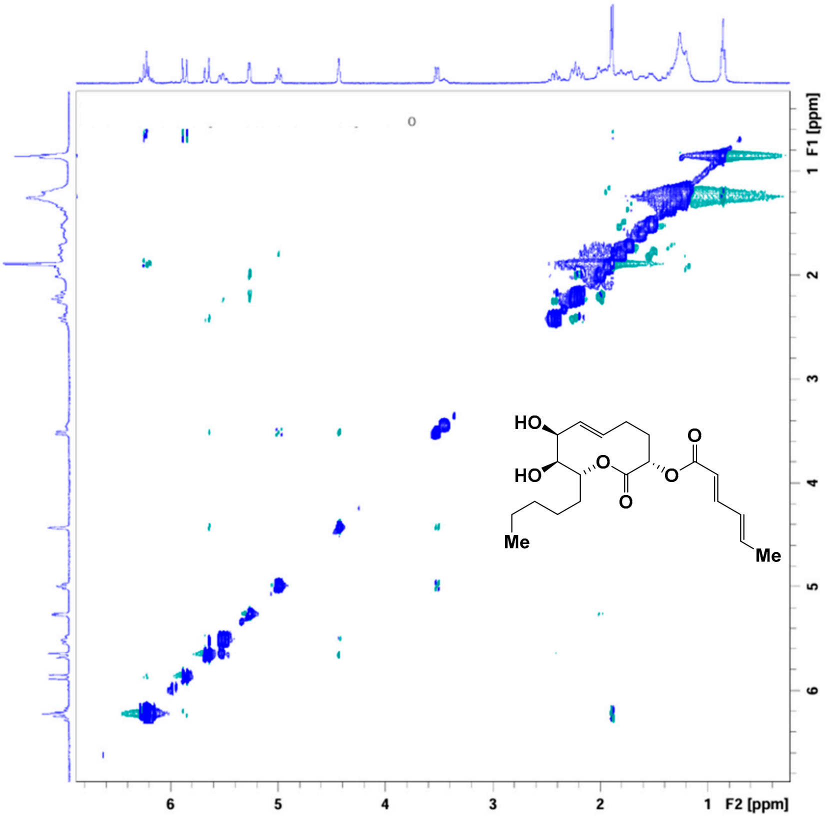

Figure S34. NOESY (400 MHz, $CDCl_3$) spectrum of bellidisin C (**2**).

2. Copies of IR Spectra

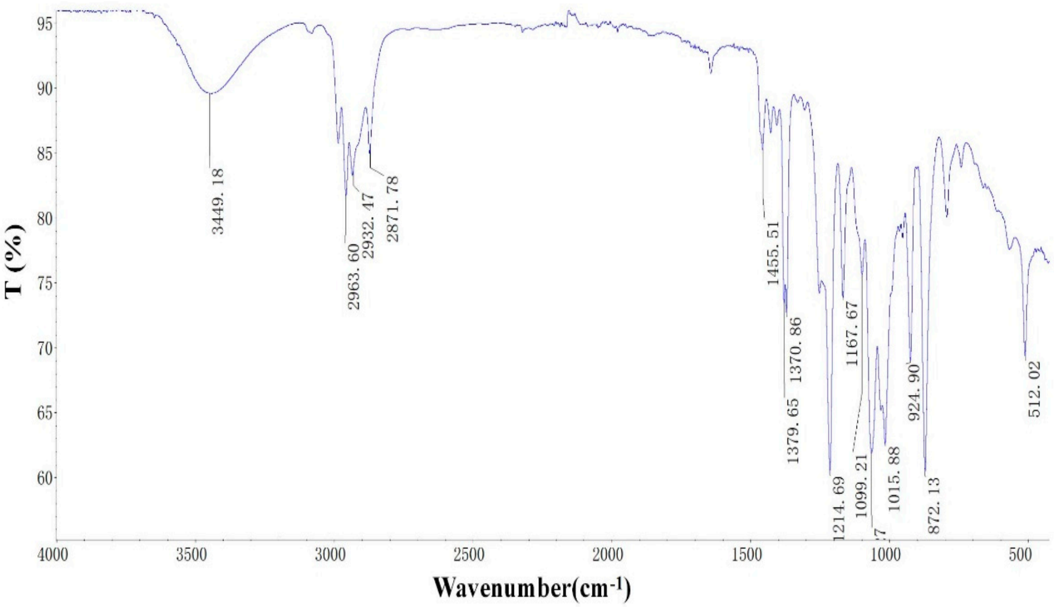

Figure S35. IR spectrum of compound **7a**.

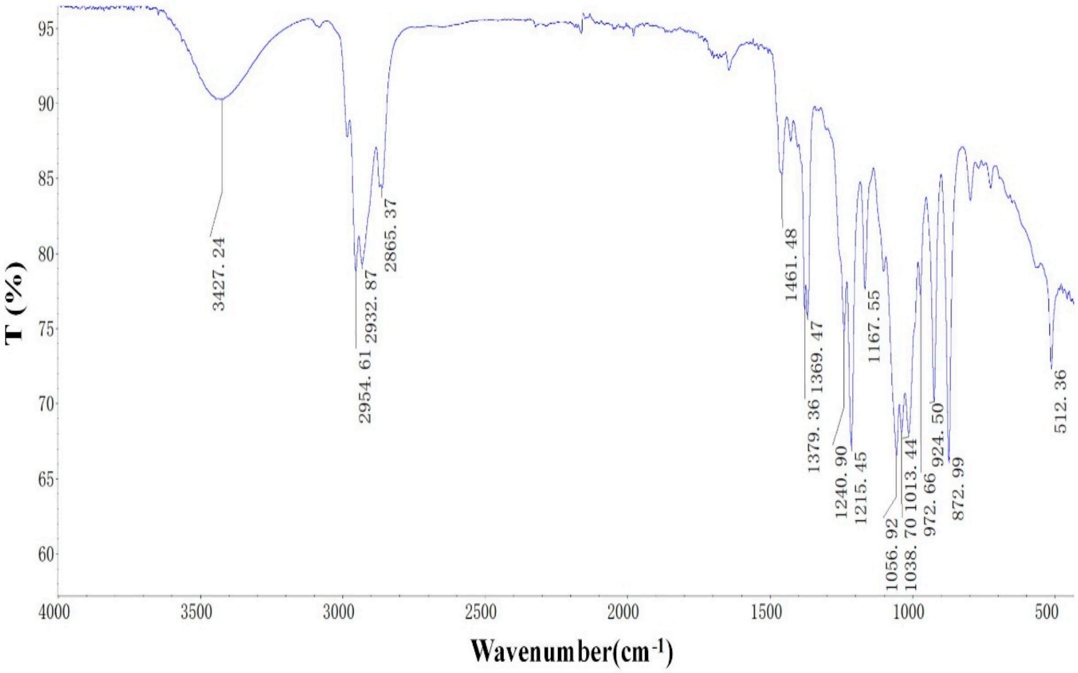

Figure S36. IR spectrum of compound **7b**.

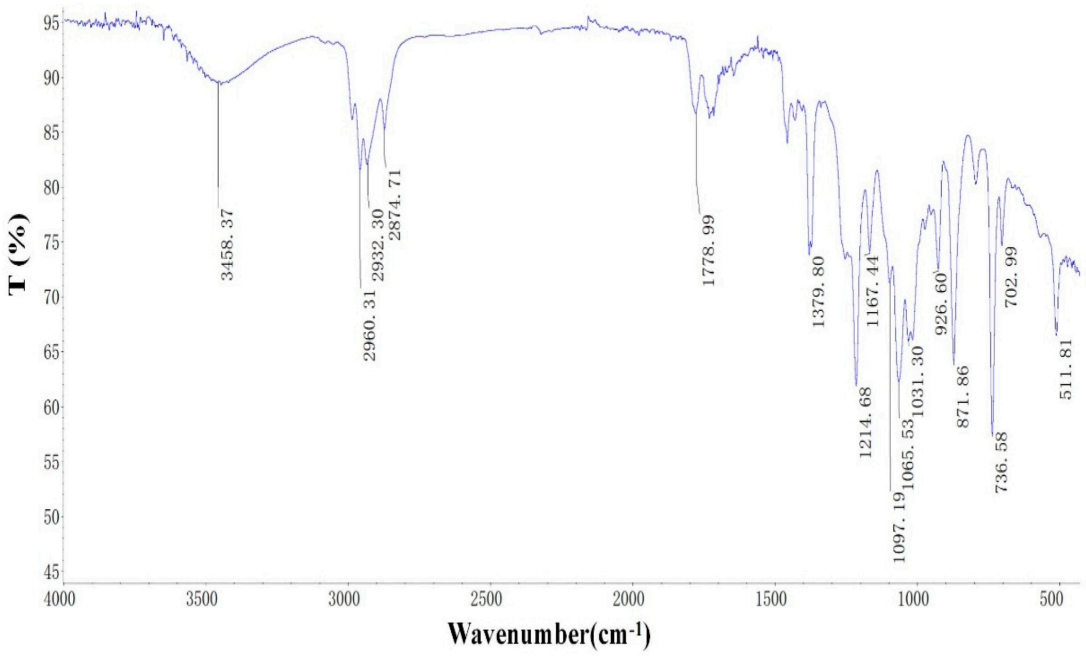

Figure S38. IR spectrum of compound **6a**.

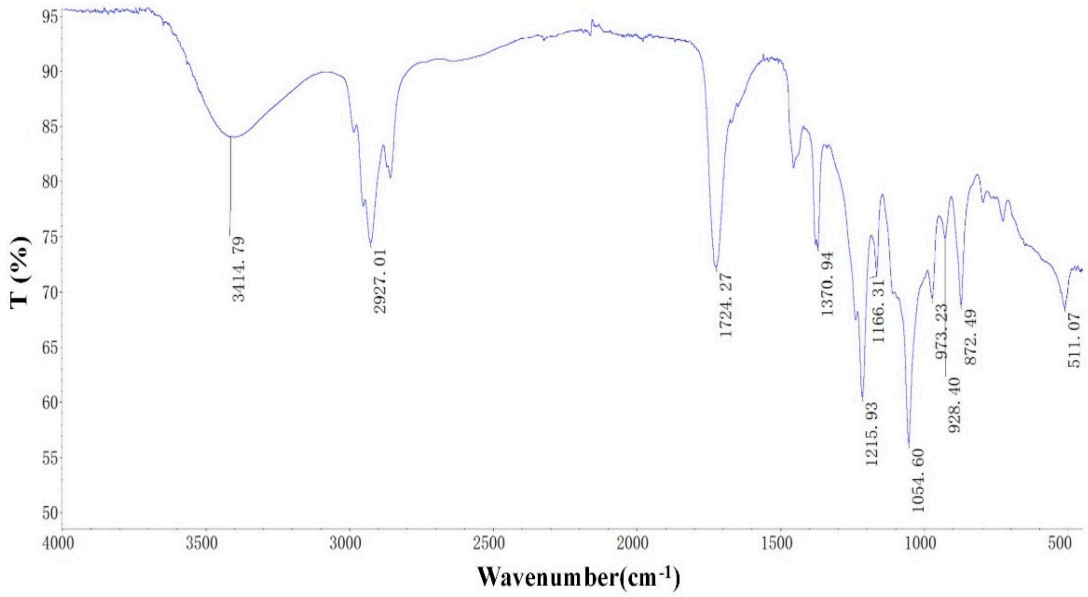

Figure S39. IR spectrum of compound **16a**.

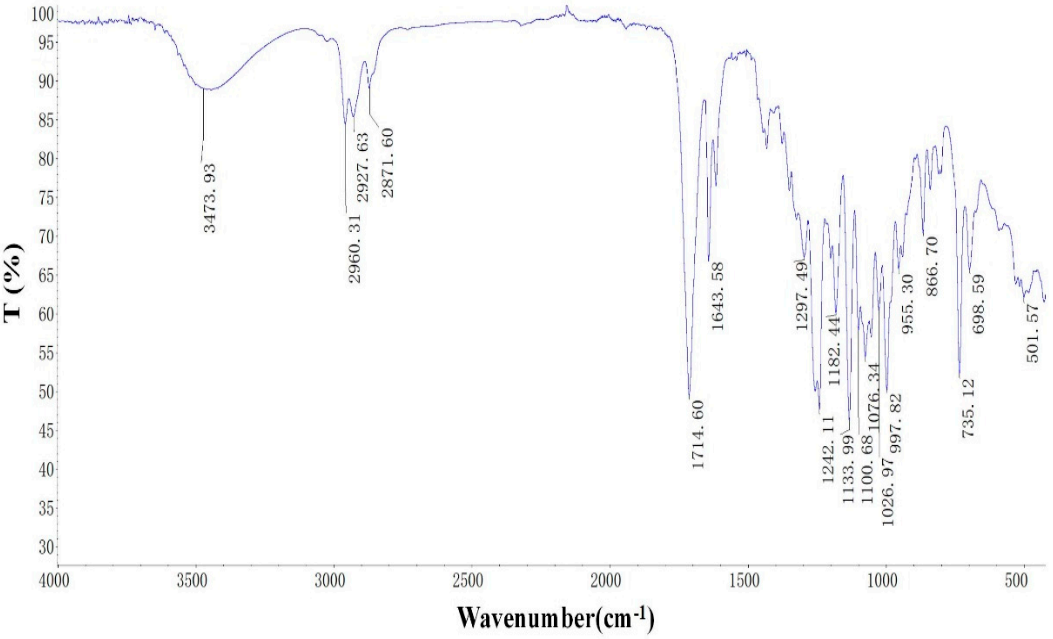

Figure S40. IR spectrum of Pinolidoxin (**1**).

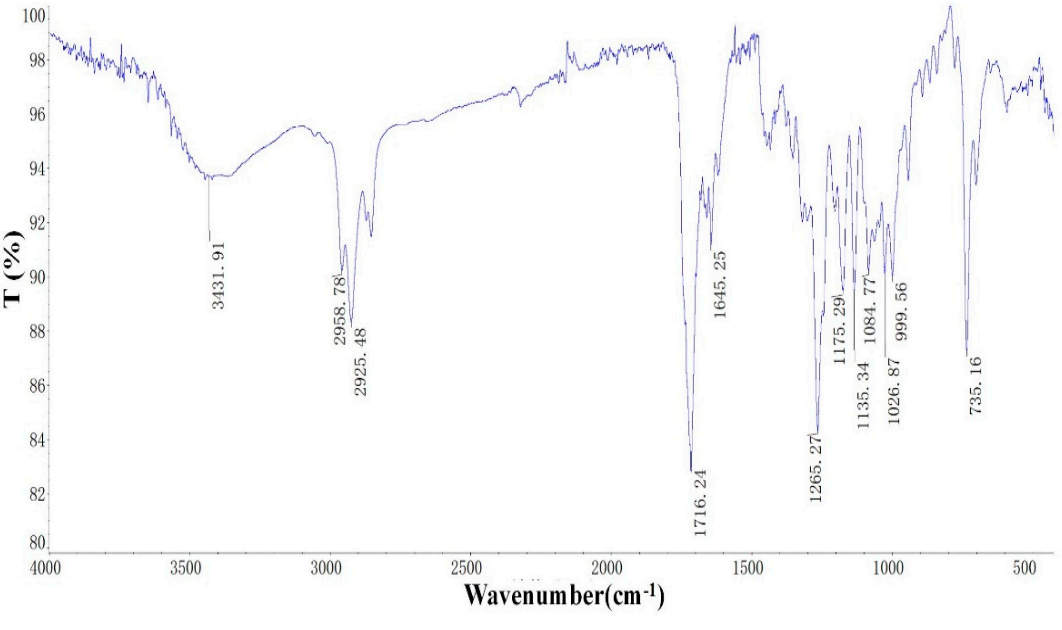

Figure S37. IR spectrum of **14a** (Z isomer of pinolidoxin).

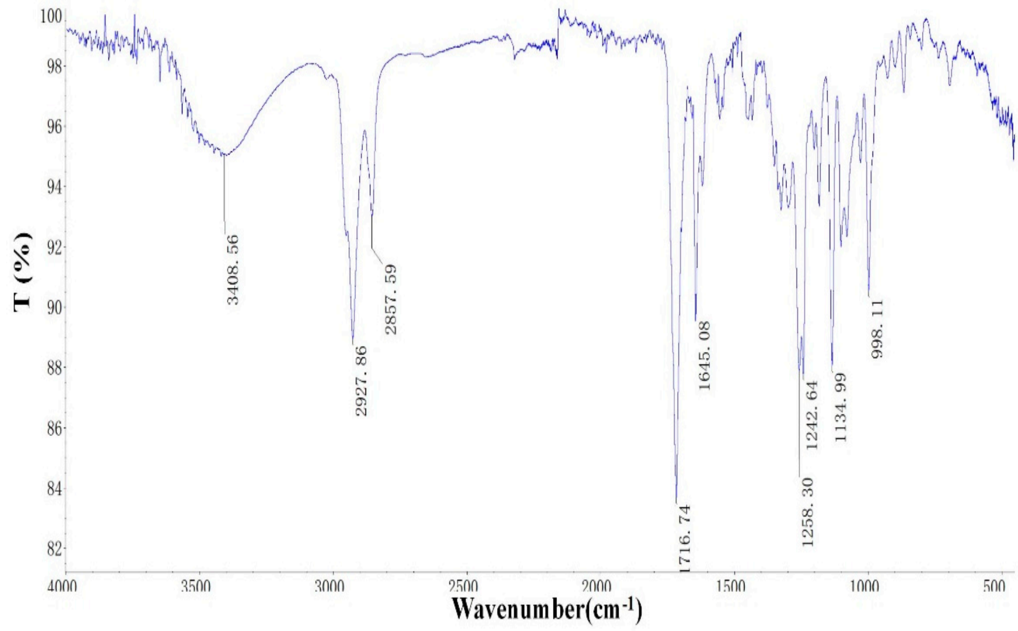

Figure S41. IR spectrum of Bellidisin C (**2**).

3. Comparison of ^{1}H and ^{13}C NMR data of pinolidoxin (**1**) and bellidisin C (**2**)

Table S1. Comparison of ^{1}H NMR [a] data of natural and synthetic pinolidoxin (**1**).

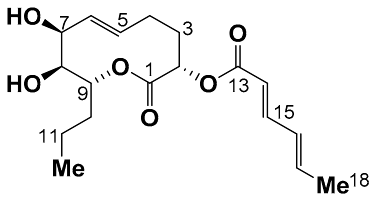

Pinolidoxin (1)

No	Natural *b*	Synthetic (Marco, J. A.) c	Synthetic (Our work)	$\Delta\delta = \delta^b$-δ^c
1	-	-	-	
2	5.25 dd (5.6, 1.7)	5.26 dd (5.4, 1.8)	5.26 dd (5.8, 1.8)	0
3	2.20 m	2.25-2.15 m	2.26-2.16 (m)	-
	2.00 m	2.00 m	2.08-2.00 (m)	
4	2.20 m	2.25-2.15 m	2.26-2.16 m	-
	2.41 m	2.42 m	2.43-2.40 m	
5	5.53 td (15.8, 15.8, 1.4)	5.53 dddd (15.8, 10.7, 4, 2.2)	5.53 td (15.6, 7.4)	0
6	5.66 dd (15.8, 1.4)	5.66 dd (15.8, 1.4)	5.65 dd (15.6, 1.4)	0.01
7	4.44 *br* s	4.44 *br* s	4.44 *br* s	0
8	3.52 dd (9.4, 2.5)	3.52 m	3.52 dd (10.0, 2.4)	0
9	5.05 td (9.4, 2.6)	5.04 td (9.4, 2.7)	5.04 td (9.4, 2.8)	0
10	1.78 m	1.78 m	1.88-1.78 (m)	-

	1.50 m	1.51 m	1.53-1.49 (m)	
11	1.33 m	1.33 m	1.37-1.31 (m)	-
	1.22 m	1.24 m	1.25-1.21 (m)	
12	0.87 t (7.3)	0.87 t (7.3)	0.87 t (7.2）	0
14	5.87 d (15.4)	5.87 d (15.4)	5.88 d (15.4)	-0.01
15	7.32 *br* dd (15.4, 9.8)	7.30 dd(15.3, 9.9)	7.31 (15.4, 9.6)	-0.01
16	6.30 m	6.30 m	6.30-6.21 (m)	-
17	6.20 m	6.25 m		-
18	1.89 br d (5.5)	1.89 d (5.8)	1.89 d (5.6)	0

[a] ^{1}H NMR spectra were recorded at 400 MHz in $CDCl_3$.

[b] Ref 6a: *Phytochemistry* **1993**, *34, 999–1003.*

[c] Ref 11c: *J. Org. Chem*. **2005**, *70*, 9822−9827.

Table S2. Comparison of ^{13}C NMR data of natural and synthetic pinolidoxin (**1**).

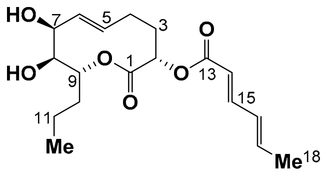

Pinolidoxin (1)

No	Natural [b]	Synthetic (Marco, J. A.) [c]	Synthetic (Our work)	$\Delta\delta = \delta^b$-δ^c
1	171.9	171.9	171.9	0
2	69.8	69.8	69.7	0.1
3	29.8	29.8	29.8	0
4	27.4	27.4	27.4	0
5	122.8	122.9	123.0	-0.1
6	132.6	132.4	132.3	0.1
7	72.9	73.0	73.1	-0.1
8	73.0	73.1	73.2	-0.1
9	71.3	71.3	71.3	0
10	33.6	33.6	33.6	0
11	17.4	17.4	17.4	0
12	13.9	13.9	13.8	0.1
13	166.1	166.1	166.0	0.1
14	118.1	118.1	118.1	0
15	145.9	145.9	145.9	0
16	129.7	129.7	129.7	0

17	140.3	140.2	140.3	-0.1
18	18.7	18.7	18.7	0

[a] ^{13}C NMR spectra were recorded at 100 MHz in $CDCl_3$.

[b] Ref 7a: *Phytochemistry* **1993**, *34,* 999–1003.

[c] Ref 11c: *J. Org. Chem*. **2005**, *70*, 9822−9827.

Table S3. Comparison of ^{1}H NMR [a] data of natural and synthetic bellidlsin C (**2**).

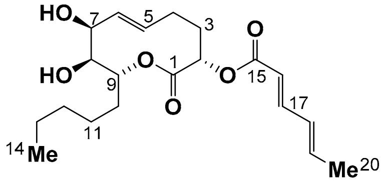

Bellidisin C (2)

No	^{1}H-natural [a]	^{1}H-synthetic (our work) [b]	$\Delta\delta = \delta^a$-δ^b
1	-	-	
2	5.26 br d (5.0)	5.26 dd (5.4, 2.0)	0
3	2.18 m	2.26-2.18 m	-
	1.99 m	2.03-1.99 m	
4	2.42 br dd (24.0, 13.0)	2.44-2.39 m	-
	2.24 m	2.26-2.18 m	
5	5.52 br dd (15.7, 13.0)	5.54-5.52 m	-
6	5.65 br d (15.7)	5.66 dd (15.6, 2.0)	-0.01
7	4.44 br s	4.43 br s	0.01
8	3.52 br d (10.0)	3.52 dd (10.0, 2.6)	0
9	5.00 br dd (10.0, 10.0)	5.00 td (10.0, 2.8)	0
10	1.52 m	1.52-1.48 m	-
	1.80 m	1.82-1.78 m	
11	1.27 overlapped	1.38-1.22 m	-
12	1.26 overlapped	1.38-1.22 m	-
13	1.26 overlapped	1.38-1.22 m	-
14	0.85 t (6.3)	0.85 t (6.4)	0
15	-	-	
16	5.87 d (15.4)	5.87 d (15.4)	0
17	7.31 dd (15.4, 10.0)	7.31 dd (15.4, 10.0)	
18	6.25 m	6.25-6.20 m	-
19	6.20 m	6.25-6.20 m	-
20	1.89 d (6.0)	1.89 d (6.0)	0

[a] ^{1}H NMR spectra were recorded at 600 MHz in $CDCl_3$. (Ref 6: *Phytochem. Lett.* **2019**, *29*, 41–46.)

[b] ^{1}H NMR spectra were recorded at 100 MHz in $CDCl_3$.

Table S4. Comparison of ^{13}C NMR data of natural and synthetic bellidlsin C (**2**).

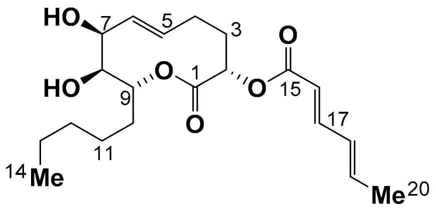

Bellidisin C (2)

No	Natural [a]	Synthetic (Our work) [b]	$\Delta\delta = \delta a\text{-}\delta b$
1	172.0	172.0	0
2	69.7	69.7	0
3	29.9	29.9	0
4	27.4	27.4	0
5	123.0	123.1	-0.1
6	132.3	132.3	0
7	73.1	73.1	0
8	73.1	73.1	0
9	71.5	71.5	0
10	31.4	31.4	0
11	23.7	23.7	0
12	31.5	31.5	0
13	22.5	22.4	0.1
14	14.0	14.0	0
15	166.1	166.0	0.1
16	118.1	118.1	0
17	146.0	145.9	0.1
18	129.7	129.7	0
19	140.3	140.3	0
20	18.8	18.8	0

[a] ^{13}C NMR spectra were recorded at 150 MHz in $CDCl_3$. (Ref 6: *Phytochem. Lett.* **2019**, *29*, 41–46.)

[b] ^{13}C NMR spectra were recorded at 100 MHz in $CDCl_3$.
